# Study on the Residual Static and Dynamic Mechanical Properties of Rubber Concrete After Elevated Temperature

**DOI:** 10.3390/ma19173809

**Published:** 2026-09-07

**Authors:** Huidong Cao, Hao Niu, Xiufeng Wu, Jinli Wang, Qiao Zhang, Jianfeng Zhao, Yang Yu

**Affiliations:** 1 School of Civil Engineering, Qingdao University of Technology, Qingdao 266520, China; cz82355@163.com (H.C.);; 2 Multidisciplinary Center for Infrastructure Engineering, Shenyang University of Technology, Shenyang 110870, China; 3 School of Civil Engineering, Liaoning Technical University, Fuxin 123000, China; wxf19760825@163.com (X.W.); wangjinli.lntu@foxmail.com (J.W.); 4 Urban Construction Technical College, Liaoning Technical University, Fuxin 123000, China; 5 Centre for Infrastructure Engineering, School of Engineering, Western Sydney University, Penrith, NSW 2751, Australia

**Keywords:** rubber concrete, high temperature, dynamic mechanics, energy dissipation, DIF

## Abstract

**Highlights:**

- The residual compressive strength decreases with rubber content and temperature, with the 30% mixture showing a strength that is 64.6% lower than that of NC at 300 °C.
- The DIFs increase with strain rate, with higher rubber content enhancing rate sensitivity and partially mitigating thermal-induced degradation.
- The energy dissipation increases substantially with strain rate; at ~100 s^−1^, rubberized mixtures generally exhibit higher energy dissipation than NC, but this trend becomes less clear at higher strain rates (≥140 s^−1^).

**Abstract:**

To address the resource utilization of waste tires and the fire-safety concerns in engineering applications of rubber concrete (RC), this study systematically investigates the residual static and dynamic mechanical properties of RC after exposure to elevated temperatures and subsequent cooling to room temperature. Specimens are prepared by replacing fine aggregate with rubber particles at equal volume replacement ratios of 0%, 5%, 15%, and 30%. After undergoing gradient heating to target temperatures ranging from 20 °C to 300 °C, the specimens are naturally cooled to room temperature prior to testing. Subsequently, static compressive and splitting tensile tests, along with dynamic impact tests using a Split Hopkinson Pressure Bar (SHPB), are performed. These experiments are supplemented by scanning electron microscopy (SEM) to elucidate the microscale mechanisms. The results show that the residual static strength decreases monotonically with increasing rubber content and temperature. For the 30% rubber content mixture, the compressive strength decreased by approximately 40.6% from ambient temperature to 300 °C, and its strength is 64.6% lower than that of NC at 300 °C. Dynamic strength exhibits a pronounced strain-rate effect, with the strain-rate sensitivity of DIF being enhanced by higher rubber content. Energy dissipation increases substantially with strain rate; rubberized mixtures generally exhibit higher energy dissipation than NC at lower strain rates, though this effect becomes less evident at higher strain rates. These findings provide a theoretical foundation for the application of RC in complex thermo-mechanical loading scenarios, particularly in evaluating its post-fire residual load-bearing capacity.

## 1. Introduction

As the most widely used civil engineering material, concrete has made its performance optimization and sustainable development a core industry priority [1,2]. Meanwhile, over one billion waste tires are generated globally each year, roughly half of which remain unrecycled [3,4,5]. Landfilling and incineration impose significant environmental burdens [6]. Incorporating waste rubber particles into concrete to produce RC not only offers an effective route for tire valorization but also substantially mitigates the inherent brittleness and poor impact resistance of conventional concrete, imparting superior toughness and energy absorption capacity. As such, RC holds considerable promise for applications in bridge seismic design and crashworthiness protection [7,8]. Rubber content serves as a key determinant of the mechanical performance of RC [9]. The existing literature indicates that while static compressive and tensile strengths tend to decline with increasing rubber content, toughness and impact resistance are noticeably improved [10,11]; beyond a content of 15%, however, strength deterioration intensifies owing to aggravated interfacial transition zone (ITZ) defects between rubber particles and the cement paste [12,13]. In practice, engineering structures are often exposed to thermal effects that induce microstructural degradation and consequent loss of macroscopic properties [14]. For RC, the thermal response is particularly intricate: below 200 °C, rubber can improve the failure mode through elastic deformation, yet once the critical temperature is exceeded, rubber decomposition triggers a marked increase in porosity and microcracking, leading to a pronounced strength reduction [15,16]. Meanwhile, elevated temperatures cause decomposition of hydration products in the cement paste, which interacts with ITZ defects to further exacerbate material damage [17,18]. In terms of macroscopic mechanical properties, YU et al. [19] have systematically investigated the residual static mechanical properties of crumb rubber concrete after elevated-temperature exposure, establishing a clear understanding of strength degradation and stress–strain curve evolution at temperatures up to 700 °C. FADIEL [20] evaluated the residual mechanical properties of rubberized concrete with 5–20% crumb rubber content after exposure to 200 °C, 400 °C, and 600 °C, revealing that specimens with 5–15% rubber content exhibited less reduction in compressive strength than conventional concrete (43–48.5% loss) in the 200–400 °C range, while splitting tensile strength is more sensitive to elevated temperatures than compressive strength.

Regarding loading patterns, engineering structures are concurrently subjected to static loads and dynamic actions such as seismic excitations, impacts, and blasts [21]. Du et al. [22] systematically investigated the effects of rubber particle size (0.1–20 mm) and content (0–30%) on the static and dynamic mechanical behavior of RC, revealing that the DIF increases with rubber content and that the 30% rubber content mixture exhibits the optimal energy absorption capacity across all strain rates. PHAM et al. [23] investigated the dynamic compressive properties of lightweight rubberized concrete with rubber contents of 0%, 15%, and 30% using SHPB tests, demonstrating that rubberized concrete exhibits excellent impact resistance and progressive failure characteristics, with strain-rate sensitivity increasing with rubber content. Nevertheless, most prior investigations have been restricted to single-temperature or single-content settings, thus lacking a systematic elucidation of the triple interplay among temperature, rubber admixture, and loading mode [24,25,26,27]. Furthermore, for the 20–300 °C interval, critical aspects including mass-loss characteristics, strength degradation kinetics, and stress–strain constitutive relations remain insufficiently understood; moreover, neither the static–dynamic performance linkage nor a unified damage evolution framework has been established, which severely constrains reliable engineering design and hazard assessment under coupled thermo-mechanical loading scenarios.

The main contribution of the present study is a systematic static–dynamic comparative analysis, with particular emphasis on a DIF-based evaluation across different temperatures and rubber contents. This approach clarifies whether the strain-rate sensitivity of rubber concrete is enhanced or compromised by thermal damage, thereby offering a reference for post-fire impact-resistant design.

## 2. Materials and Methods

### 2.1. Raw Materials and Mix Proportions

Ordinary Portland cement (grade P·O42.5) is used as the cementitious material. Natural river sand with an apparent density of 2600 kg/m^3^ is adopted as the fine aggregate, and natural crushed stone with a particle size of 5–15 mm and an apparent density of 2680 kg/m^3^ serves as the coarse aggregate. The modified admixture is waste rubber particles (particle size 0.45–0.6 mm, bulk density 1100 kg/m^3^), which are subjected to a high-temperature mixing pretreatment following the procedure in reference to enhance their interfacial bonding with the cement paste [28]. Specifically, the rubber particles are placed in a high-temperature mixing tank and stirred at 180 °C for 15 min at a rotation speed of 700 rpm. A total of four mix groups are designed by replacing fine aggregate with rubber particles at volume replacement ratios of 0%, 5%, 15%, and 30% via the equal volume replacement method. To address the issues of particle flotation and mixture segregation caused by the strongly hydrophobic nature of rubber particle surfaces, a phased wet-coating mixing process is adopted to prepare the rubber concrete mixtures. The procedure comprises three stages: (1) Pre-wetting stage: fine aggregate and all rubber particles are placed into a forced-action mixer with 30% of the total mixing water and premixed for 30 s to thoroughly wet the rubber surfaces; (2) Coating stage: all cement and 50% of the total water are added and mixed for 50 s to form a uniform paste, ensuring that rubber particles are fully encapsulated by cement slurry; (3) Grading and forming stage: coarse aggregate and the remaining 20% of water are introduced and mixed for 80 s to achieve a homogeneous mixture and to expel entrapped air. Immediately after mixing, the concrete is cast into molds and vibrated until the surface is covered with paste and no visible air bubbles emerge. The specimens are demolded after 24 h and then cured in a standard curing chamber at (20 ± 1) °C with a relative humidity of ≥95% for 28 days prior to testing. Two types of specimens are cast using plastic molds: cubic specimens of 100 mm × 100 mm × 100 mm for quasi-static compressive tests, and disk-shaped specimens of Φ100 mm × 50 mm for static Brazilian splitting tensile tests and SHPB dynamic impact compression tests. To ensure result reliability and minimize random errors, three specimens are prepared for each mix proportion and each temperature level. Specimens are designated as follows: NC represents normal concrete, while RC followed by a numeral indicates the rubber volume replacement ratio. The specific mix proportions are summarized in Table 1.

### 2.2. Specimen Preparation and High-Temperature Treatment

**Figure 1:**
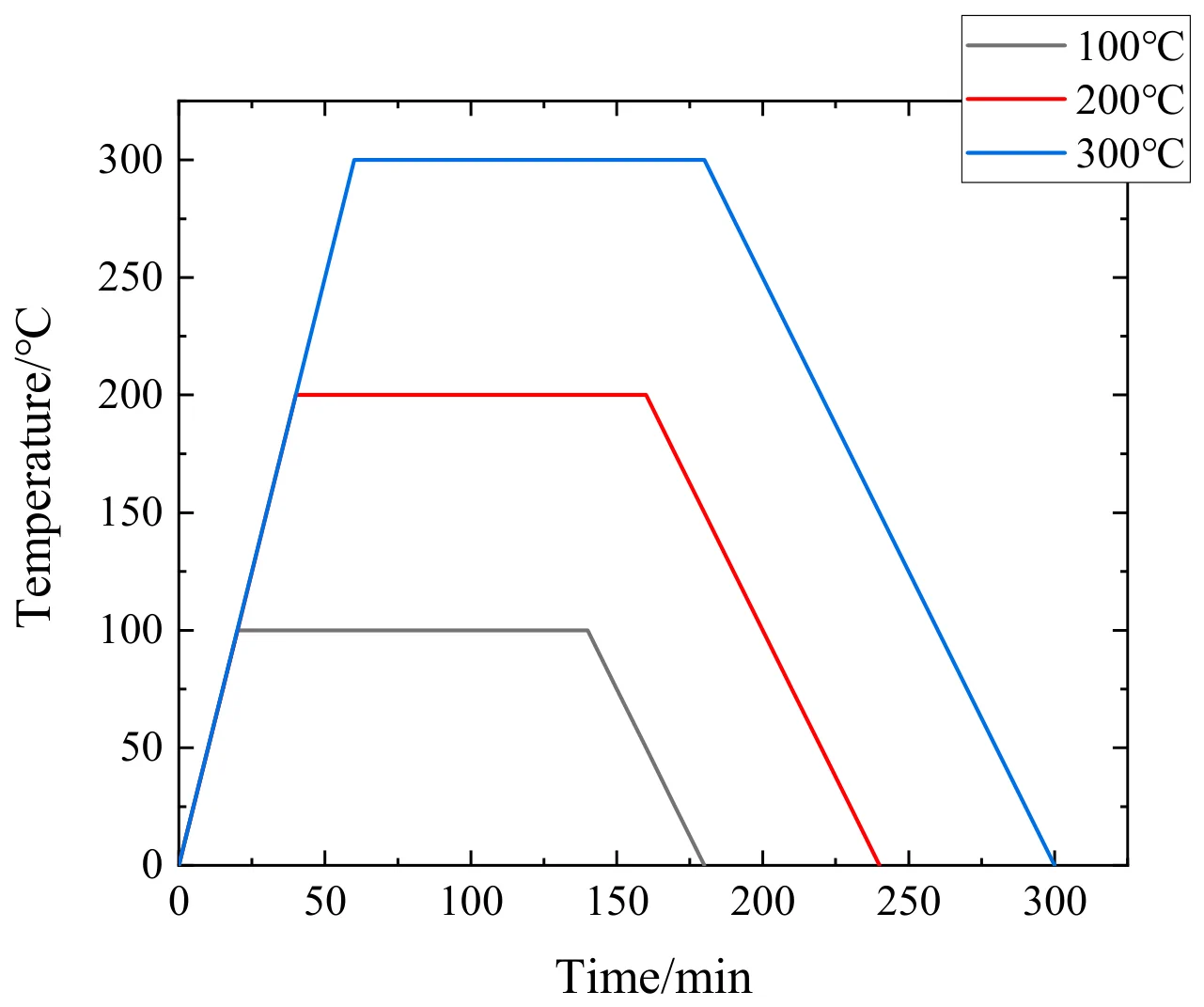
The temperature–time curve of concrete heating.

Cube specimens with dimensions of 100 mm × 100 mm × 100 mm and disk specimens with dimensions of Φ100 mm × 50 mm are prepared for the thermal exposure tests. A programmable electric muffle furnace with a maximum operating temperature of 1200 °C is employed for the gradient heat treatment. The target temperatures are set at 100 °C, 200 °C, and 300 °C. The heating procedure for the concrete specimens is established following the method of Ref. [29]. The temperature–time histories for the three exposure conditions are presented in Figure 1. The furnace is equipped with built-in K-type thermocouples positioned at the center and corners of the chamber to monitor the ambient temperature, with readings continuously recorded by a data acquisition system. Specimens are heated from ambient temperature to each target temperature at a nominal heating rate of 5 °C/min. Upon reaching the target temperature, the specimens are held for 2 h to ensure uniform temperature distribution throughout the cross-section. After the holding period, the furnace is switched off, and the specimens are cooled to room temperature at a controlled rate of 2.5 °C/min to prevent non-uniform cooling, which could otherwise induce additional thermal damage to the concrete specimens. After cooling, the apparent damage characteristics of the specimens are observed, and the mass loss is measured. Meanwhile, representative specimens are selected for sampling, and SEM is employed to observe the microstructural morphology of the ITZ.

### 2.3. Test Methods

#### 2.3.1. Static Compressive Test

**Figure 2:**
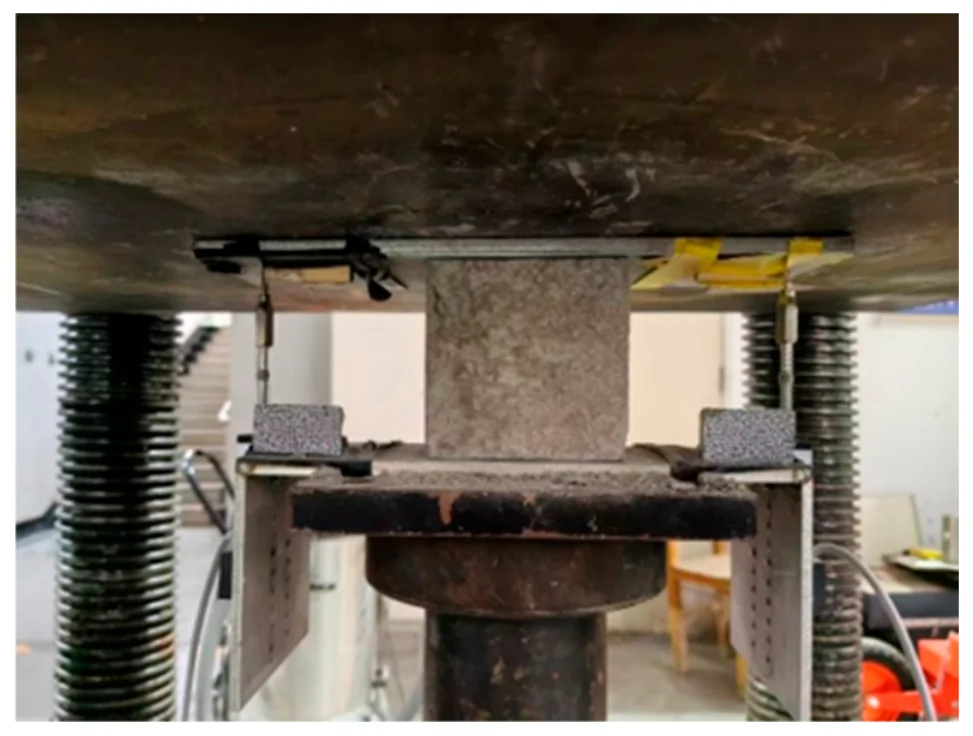
Static compressive test of concrete.

An electro-hydraulic servo pressure testing machine is used to conduct static compressive performance tests on RC specimens after different temperature treatments; the testing machine and loading process are shown in Figure 2. A vernier caliper is used to accurately measure the actual side lengths of the specimens, and the average values are taken to calculate the bearing cross-sectional area. Basic information such as rubber content and treatment temperature is recorded simultaneously, and qualified specimens without corner defects, cracks, or performance inhomogeneity are screened for testing. The quasi-static experiments follow the Chinese standard (GB/T50081-2019) [30]. The loading rates for the static compressive strength test and the static splitting tensile test of concrete are 0.5 MPa/s and 0.05 MPa/s, respectively.

#### 2.3.2. Static Splitting Tensile Test

**Figure 3:**
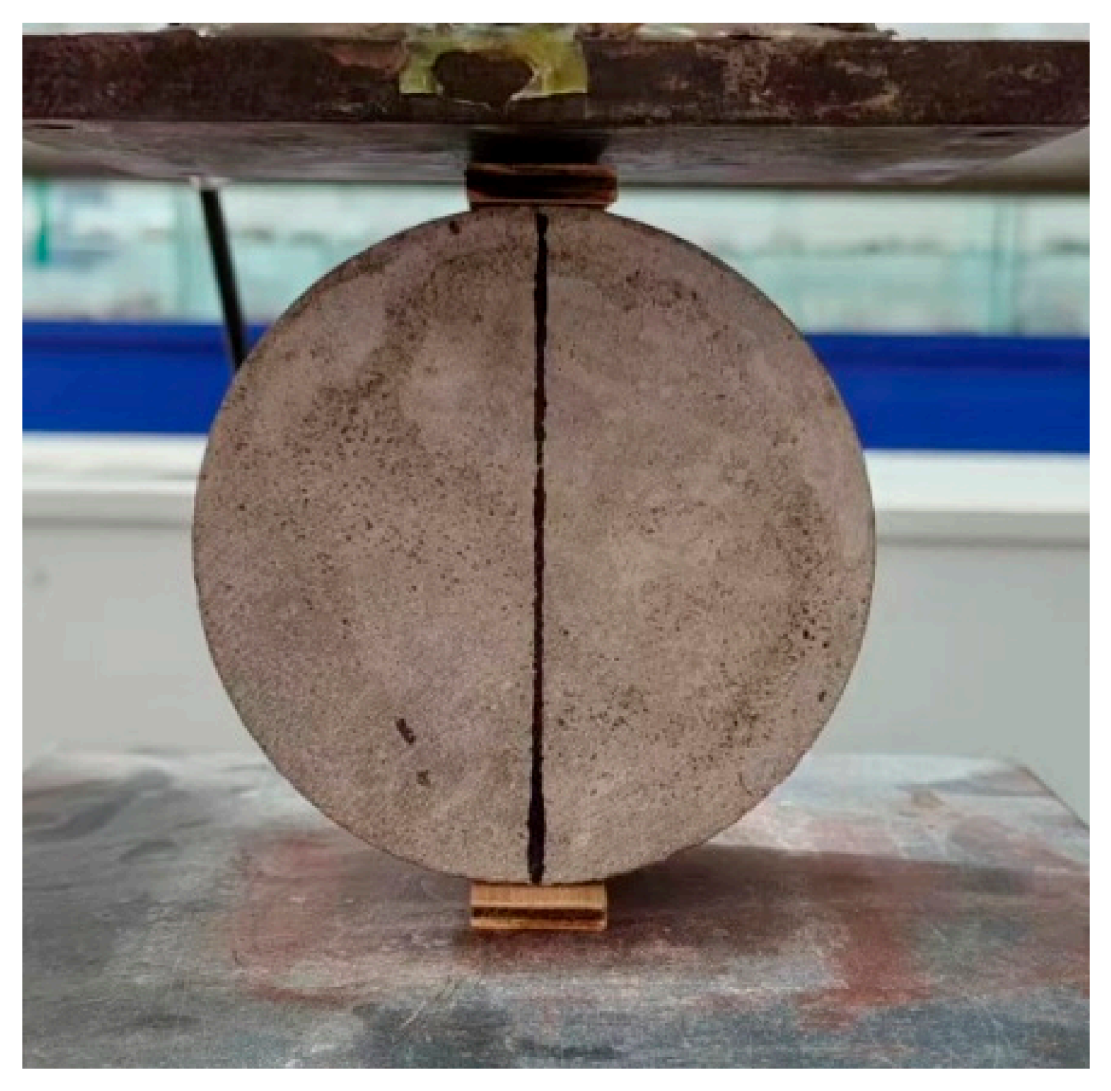
Splitting tensile test of concrete.

This test employs disk specimens with a diameter of 100 mm and a thickness of 50 mm, as shown in Figure 3. After curing, the end faces of the specimens are ground and polished to ensure surface flatness and parallelism, meeting the SHPB test requirements. Three parallel specimens are prepared for each combination of rubber content and temperature. Before testing, qualified specimens with dimensional deviations within ±1 mm and no visible surface cracks are selected.

It should be noted that the RC30 mixture is excluded from the splitting tensile tests across all temperature levels. The exclusion is based on the following considerations. First, after exposure to 300 °C, a portion of the RC30 disk specimens exhibit visible corner breakage and localized surface damage, and these damaged specimens are excluded from further testing. Second, for the remaining RC30 specimens that survive the 300 °C exposure, local cracking is observed during the preloading stage of the splitting tensile tests, due to the inherently low dynamic tensile strength of concrete. This preloading damage further compromises the integrity of the specimens. In addition, the splitting tensile strength results for RC30 after exposure to 200 °C and 300 °C show considerable scatter, making it difficult to draw reliable conclusions from the limited valid data. Therefore, to ensure the reliability and comparability of the experimental results, RC30 is not included in the splitting tensile test program. Consequently, the corresponding discussions are confined to the NC, RC5, and RC15 mixtures actually tested.

#### 2.3.3. Dynamic Compressive Test

**Figure 4:**
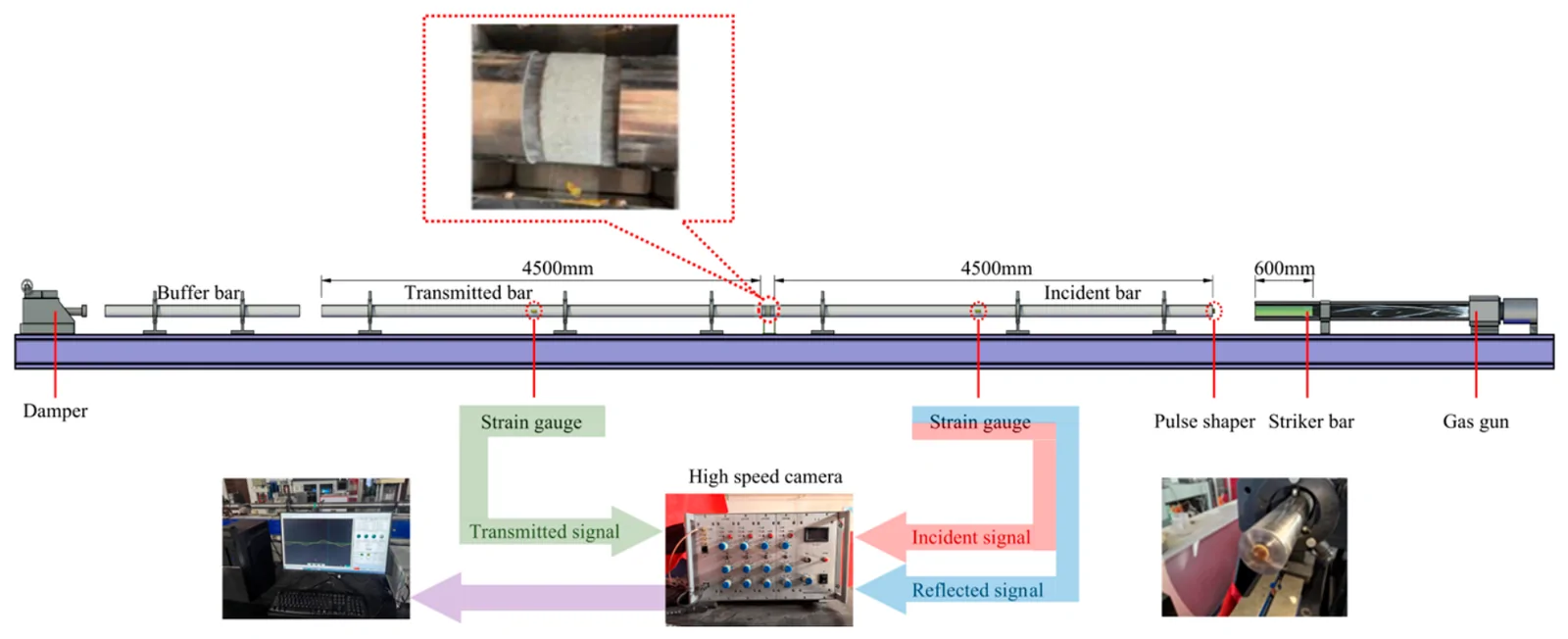
Concrete dynamic impact test.

A Φ100 mm SHPB system is employed to test the dynamic mechanical properties of RC after high-temperature exposure, with the schematic diagram of the apparatus shown in Figure 4. The incident and transmitted bars are both made of high-strength steel with a diameter of 100 mm and a length of 4500 mm for each bar. The elastic modulus of the bar material is 206 GPa, the stress wave velocity is 5190 m/s, and the density is 7850 kg/m^3^. By adjusting the impact air pressure, three distinct impact velocities are applied to the specimens. To minimize friction at the bar–specimen interfaces, a thin layer of lubricant is uniformly applied to both ends of each specimen prior to testing. The strain gauges are all attached in the middle of the incident bar and the transmitted bar. The acquisition frequency of the signal acquisition system is 10 MHz. To determine the appropriate pulse shaper, preliminary comparative tests are conducted using brass disks with diameters of 15 mm and 30 mm and thicknesses of 1 mm and 2 mm under different impact pressures. The selection criteria are based on the principle that the pulse shaper should be capable of extending the rise time of the incident wave to ensure stress equilibrium and a nearly constant strain rate during loading, while maintaining a transmitted signal sufficiently above the noise level. The final loading parameters and the size of the pulse shaper are presented in Table 2. For each test condition, 3 specimens are tested to ensure statistical reliability. The reported variations in dynamic test results represent standard deviations calculated from these replicate measurements.

## 3. Apparent and Microscopic Characteristics of Specimens

### 3.1. Apparent Characteristics

Real-time observations conducted during the high-temperature heating process indicated that the apparent features of the specimens evolved in a regular manner as the temperature rose. At 200 °C, pungent gases were detected escaping from the furnace; at 300 °C, conspicuous white smoke emerged, accompanied by a strong, irritating odor. These phenomena are ascribable to the volatilization and exudation of low-molecular-weight additives (e.g., plasticizers and anti-aging agents) from the rubber particles, along with the release of gaseous pyrolysis products generated from the scission of rubber molecular chains.

**Figure 5:**
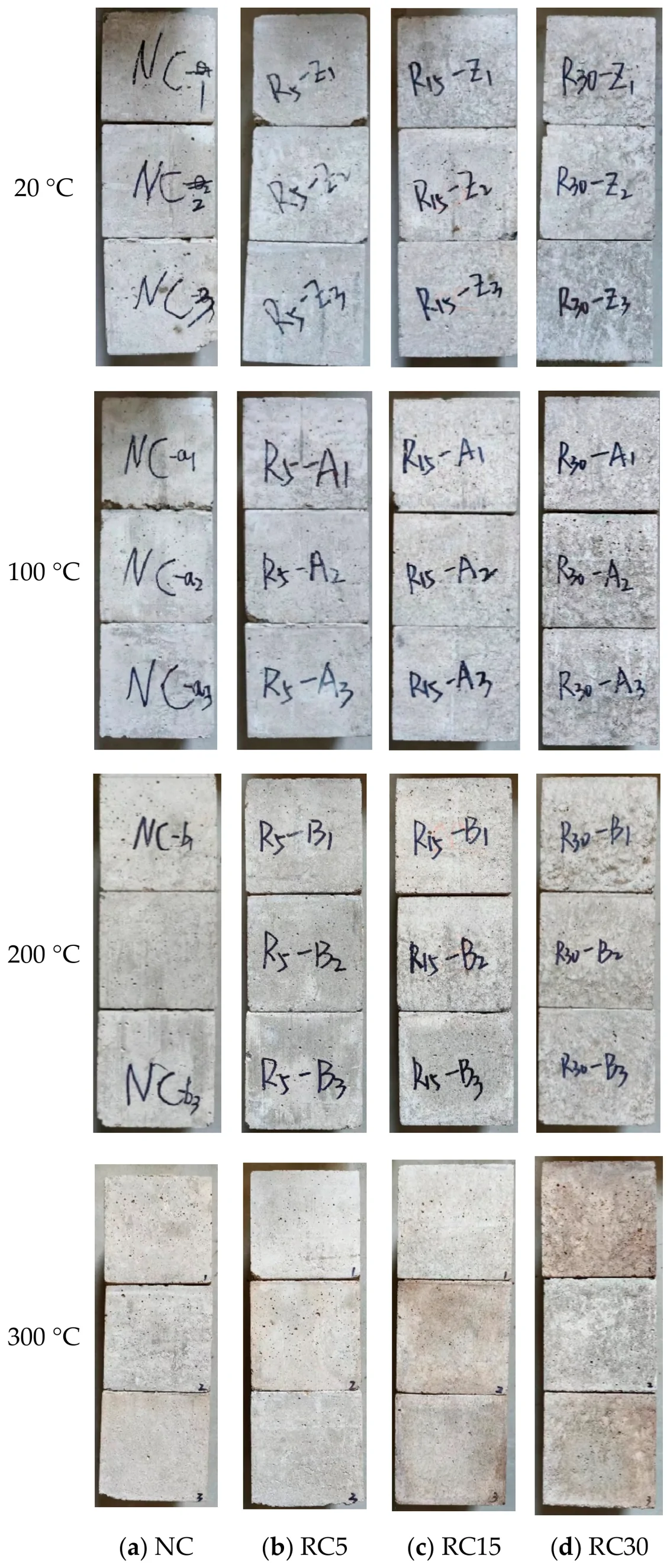
The apparent characteristics of concrete.

Figure 5 presents the apparent morphological variations in specimens with different rubber contents following exposure to various temperatures. After treatment at 100 °C, the specimen surfaces become slightly lighter in color with localized whitish areas, attributable to the evaporation of internal free water, while the overall structural integrity remains intact. Following treatment at 200 °C, the surface color further faded, and the rubber particles softened and melted. Given that the coefficient of thermal expansion (CTE) of rubber (approximately 6 × 10^−4^–8 × 10^−4^/°C) differs from that of the cementitious matrix (approximately 1 × 10^−5^–1.5 × 10^−5^/°C) by roughly one order of magnitude, distinct microcracks emerged in the ITZ. After exposure to 300 °C, the specimens turned reddish-brown with local color non-uniformities, which are the result of the synergistic action of iron oxide coloration (induced by evaporation of chemically bound water), decomposition of hydration products, and thermal degradation of the rubber. Macroscopic cracks caused by thermal stresses are observable on the surfaces; these cracks tended to propagate and coalesce preferentially along the rubber–paste interfaces and existing micropores. Consequently, interfacial bonding is severely compromised, the compactness of the specimens decreased, and the porosity increased substantially. However, the coarse aggregate skeleton remained essentially intact, and the concrete did not experience any disruptive collapse at the macroscopic level.

### 3.2. Microscopic Characteristics

**Figure 6:**
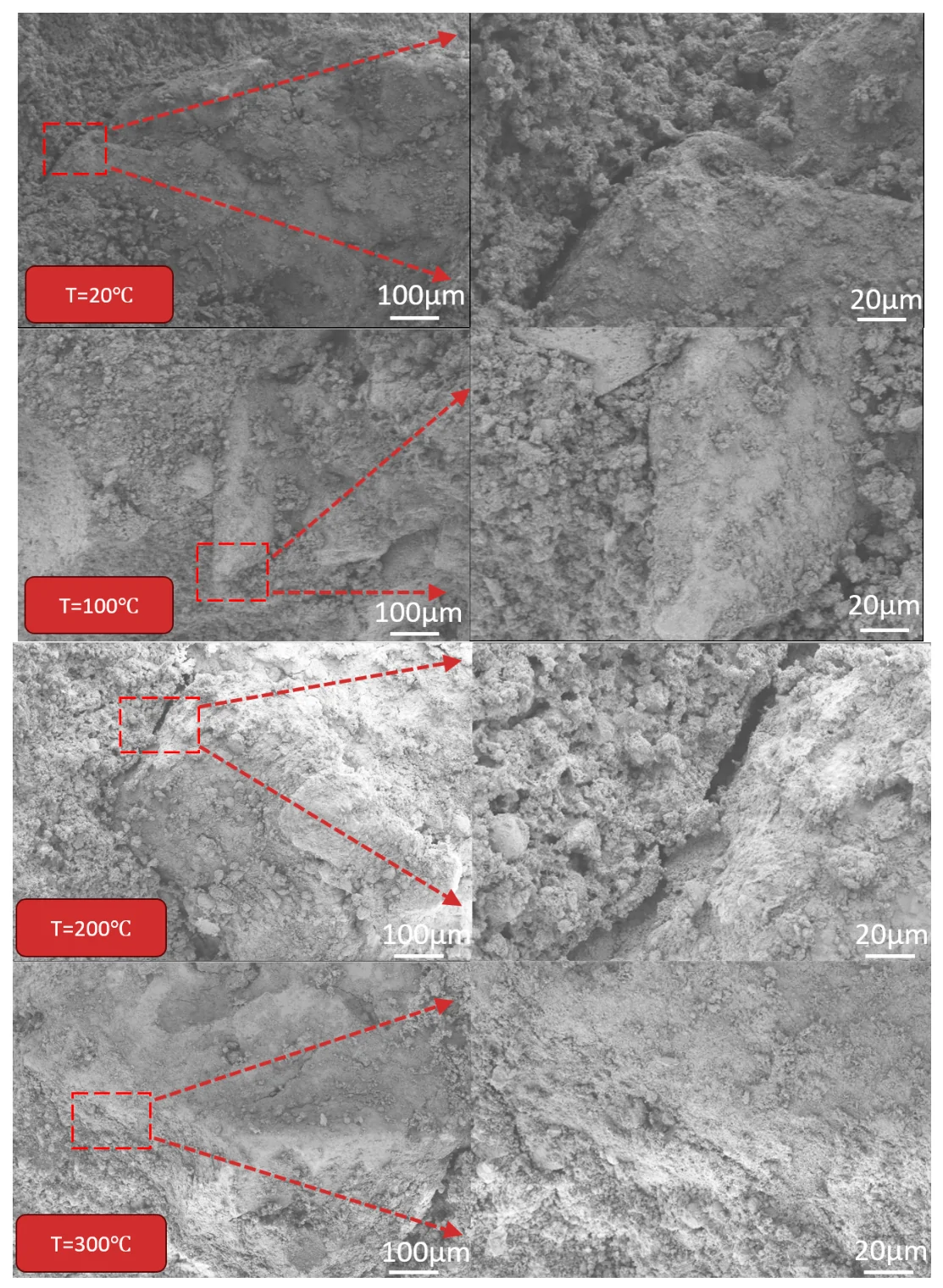
SEM images of the ITZ of RC.

To intuitively reveal the microstructural evolution and damage characteristics of rubber particles, the cement paste matrix, and the ITZ at different temperatures. SEM observations are conducted on the fracture surfaces of RC specimens, with particular attention paid to the rubber particles and their adjacent interfacial zones across the four target temperature levels. As evident from Figure 6, at ambient temperature (20 °C), noticeable gaps existed between the rubber particles and the cementitious matrix. These gaps account for the generally lower compressive and splitting tensile strengths of RC relative to ordinary concrete at room temperature. Following treatment at 100 °C, the strength loss pattern remained largely comparable to that observed at ambient temperature. However, since 100 °C approaches the boiling threshold of both free water and capillary water within the concrete, partial moisture evaporation led to a slight reduction in strength compared with the untreated specimens.

When the temperature is elevated to 200 °C, the demarcation between rubber particles and the cementitious matrix becomes less distinct. This blurring is attributable to the fact that 200 °C corresponds to the incipient stage of thermo-oxidation and thermal degradation of rubber, at which point the structural integrity of the rubber particles begins to deteriorate. A similar phenomenon has been reported in Ref. [28], where the onset of rubber thermal decomposition is observed at temperatures above 200 °C. At 300 °C, the performance degradation of RC reached its peak severity. The rubber particles underwent pronounced thermal decomposition, the hydration product structure of the cement pastes further collapsed, and the rubber–matrix interface grew increasingly obscure. The intense thermal degradation of the rubber particles not only completely nullified their toughening effect but also transformed them into the predominant internal damage source within the concrete. This mechanistic transition fundamentally explains why the performance deterioration of RC at 300 °C substantially surpasses that of NC.

## 4. Results and Discussion

### 4.1. Mass Loss

The mass loss rate serves as a key macroscopic indicator for quantitatively assessing the extent of high-temperature damage, as it integrates the combined contributions of internal moisture evaporation, decomposition of hydration products, and thermal degradation of organic constituents. The mass loss rate is determined using Equation (1):

(1) $$M=\frac{m_{0}-m_{1}}{m_{0}}\times 100\%$$

where M denotes the mass loss rate (%), $m_{0}$ is the specimen mass at ambient temperature (g), and $m_{1}$ is the specimen mass following high-temperature treatment (g).

**Figure 7:**
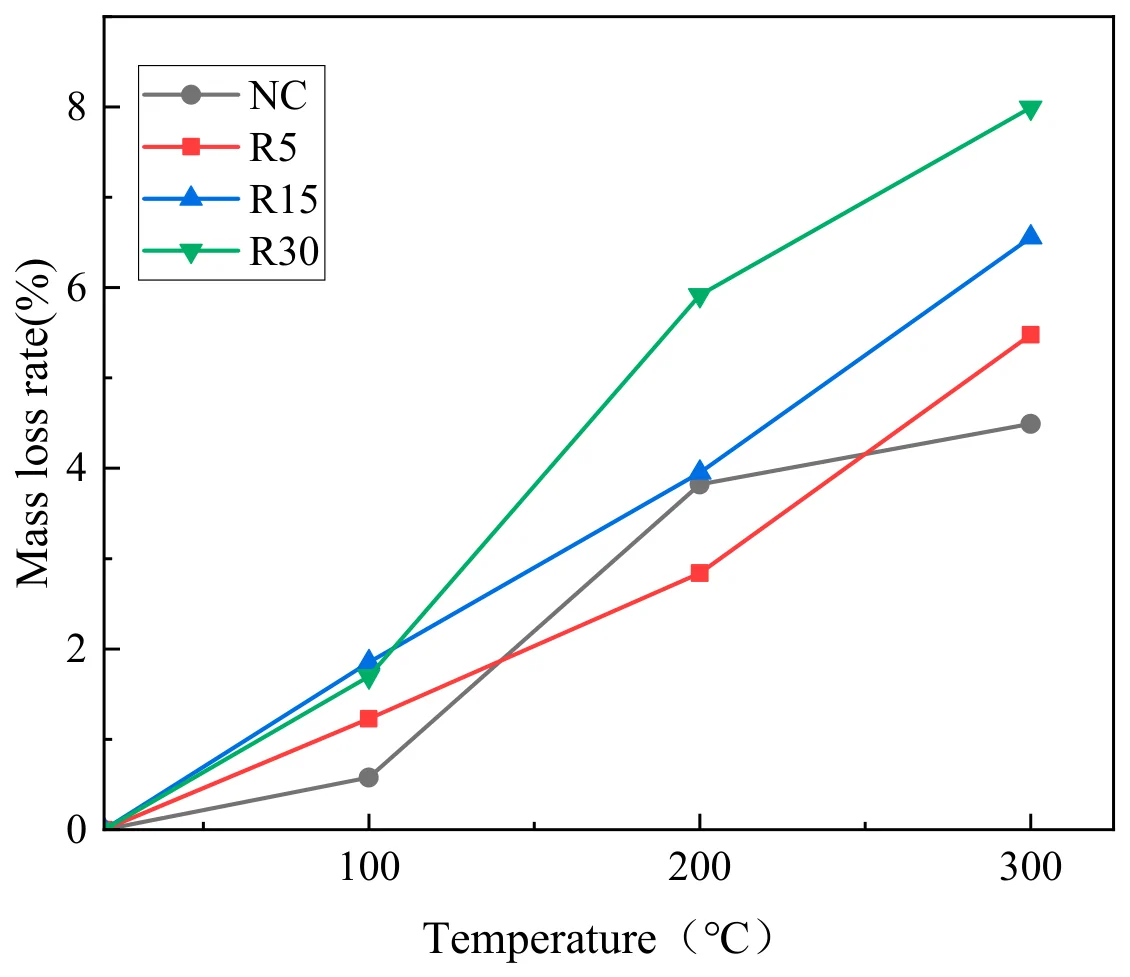
Mass loss curves of cubic RC specimens at different temperatures.

Figure 7 shows the mass loss rate curves for cubic specimens with varying rubber contents subjected to different temperature levels. The results demonstrate that the mass loss rate of all mixtures increased monotonically with elevated temperature; nonetheless, the increment magnitude across distinct temperature stages and the modulating effect of rubber content differed considerably. In stage I (20–100 °C), the mass loss rate rose mildly, with increments falling within 0.5–1.8% for all specimens. The mass loss at this stage is predominantly caused by free water evaporation, while the rubber content exerted only a marginal influence. In stage II (100–200 °C), the mass loss rate accelerated notably, with increments reaching 2.8–5.9%. Beyond the ongoing evaporation of residual free water, cement hydration products—chiefly ettringite and C-S-H gel began to dehydrate and decompose. Concurrently, low-molecular-weight volatiles (e.g., plasticizers and antioxidants) started to exude from the rubber particles in minor quantities. The effect of rubber content became substantially more pronounced during this stage: higher rubber content introduced more thermally labile components, thereby compromising mass stability. In stage III (200–300 °C), the mass loss escalated further, with the RC30 mixture reaching 7.99%. At this stage, the dehydration of cement hydration products continued to progress. More critically, the rubber polymer backbones underwent thermal degradation (the onset pyrolysis temperature of natural rubber is approximately 250 °C), leading to extensive chain scission and the release of low-molecular-weight hydrocarbon volatiles [20]. The synergistic interplay between hydration product decomposition and rubber thermal degradation drove an accelerated rise in mass loss.

### 4.2. Static Mechanical Properties of Concrete

#### 4.2.1. Static Compressive Strength

Table 3 presents the compressive strength evolution of cubic RC specimens with rubber replacement ratios of 0%, 5%, 15%, and 30% as a function of exposure temperature. With increasing temperature, the compressive strength of rubber concrete decreases for all rubber contents, and higher rubber content leads to more pronounced loss. At 20 °C, the strengths of NC, RC5, RC15, and RC30 are 53.63, 46.36, 36.92, and 29.18 MPa, respectively. In the 20–100 °C range, the reduction remained limited. The compressive strength of NC dropped from 53.6 MPa to 50.95 MPa (5.0% loss), while RC30 decreased from 29.18 MPa to 22.57 MPa (22.7% loss). Between 100 and 200 °C, the decline becomes more pronounced, with NC decreasing to 50.00 MPa and RC30 to 19.05 MPa. In the 200–300 °C range, the most pronounced degradation is observed, with NC falling to 49.98 MPa and RC30 to 17.32 MPa. Overall, from 20 °C to 300 °C, the total reduction in compressive strength is 8.6% for NC and 40.6% for RC30. The negative effect of rubber content on compressive strength is amplified by temperature: at 20 °C, the strength of RC30 is 45.6% lower than that of NC, while after exposure to 300 °C, this difference widened to 64.6%.

#### 4.2.2. Static Splitting Tensile Strength

Table 4 summarizes the splitting tensile strength results of concrete specimens subjected to different temperature treatments. At ambient temperature (20 °C), the splitting tensile strengths of the NC, RC5, and RC15 specimens are 6.58 MPa, 5.27 MPa, and 3.65 MPa, respectively, corresponding to reductions of 19.9% and 30.6% relative to the NC reference group. The reduction rate exhibited an accelerating trend with increasing rubber content. This behavior is primarily attributable to two factors: first, the ITZ between rubber and the cementitious matrix acts as the weakest link in the material—higher rubber content enlarges the ITZ area fraction, introduces more interfacial defects, and reduces stress transfer efficiency; second, the rubber particles are prone to plastic deformation under load, which further diminishes the overall tensile resistance.

It should be noted that the RC30 specimens are excluded from the splitting tensile test. During specimen preparation for the dynamic splitting tensile tests, the RC30 specimens exhibited visible corner breakage and localized surface damage after the 300 °C heat treatment, which would compromise the reliability and representativeness of the test results. Given that the primary objective of the dynamic tests is to evaluate the strain-rate dependent behavior under impact loading, testing specimens with pre-existing macroscopic defects would introduce additional variability that obscures the intrinsic material response. Therefore, to ensure the validity and comparability of the experimental data, the RC30 mixture is not included in the splitting tensile and SHPB test programs. Consequently, the subsequent discussions concerning the coupled effects of temperature, rubber content, and loading mode are confined to the NC, RC5, and RC15 mixtures actually tested.

Following treatment at 100 °C, the splitting tensile strengths of the NC, RC5, and RC15 specimens are 6.44 MPa, 4.94 MPa, and 3.37 MPa, with reductions of 23.3% and 31.6% relative to the NC reference. The decreasing rate remained generally similar to that observed at ambient temperature. Since this temperature level only induced free water evaporation within the concrete without noticeable damage to either the rubber particles or the rubber–cement interfaces, rubber content continued to dominate the tensile strength. Of note, the reduction for RC5 is slightly larger than that at ambient temperature, suggesting that mild heating marginally amplified the influence of interfacial defects—a phenomenon attributable to the evaporation of free water, which increased micropores at the interface and further weakened the interfacial bond strength. After exposure to 200 °C, the splitting tensile strengths of the NC, RC5, and RC15 specimens are 5.47 MPa, 4.60 MPa, and 3.09 MPa, with reductions of 16.0% and 33.2% relative to the NC reference. Notably, the decreasing rate for RC5 slowed somewhat, whereas that for RC15 accelerated substantially. This divergence can be explained as follows: at 200 °C, dehydration of cement hydration products commenced, leading to a reduction in the strength of the cement paste itself, which narrowed the strength gap between NC and RC5. For the RC15 specimens, however, thermal degradation of rubber becomes more severe, further exacerbating interfacial defects and resulting in a larger strength reduction—illustrating the combined deterioration effect of elevated temperature and high rubber content. Following treatment at 300 °C, the splitting tensile strengths of the NC, RC5, and RC15 specimens are 5.21 MPa, 4.30 MPa, and 2.74 MPa, with reductions of 18.6% and 32.1% relative to the NC reference, and the decreasing rate tended to level off. At this temperature, both cement paste dehydration and rubber thermal degradation have proceeded extensively, rendering the internal damage across different rubber contents more comparable; consequently, the diminishing effect of rubber content on tensile strength also stabilized. Nonetheless, the strength of the RC15 specimens amounted to only 52.6% of that of NC, indicating that the strength penalty associated with high rubber content becomes more conspicuous under elevated-temperature conditions.

From the standpoint of engineering applications, the splitting tensile strength of the RC5 specimens remained above 4.30 MPa across all temperature conditions—23.3–39.5% higher than that of RC15—while offering ductility superior to NC. Hence, RC5 may serve as a suitable option when a trade-off between strength and toughness is desirable. Although RC15 exhibits relatively lower tensile strength, it provides excellent ductility and energy absorption capacity, which may be advantageous in applications where tensile strength is less critical, such as seismic retrofitting and cushion layers. Final selection should depend on the specific performance demands of the target application. Under identical rubber content conditions, the splitting tensile strength decreased in a stage-dependent manner with increasing temperature. The rate of reduction varied across different temperature intervals, and higher rubber replacement levels are associated with more pronounced strength losses at elevated temperatures. For the NC specimens, the strength reductions at 100 °C, 200 °C, and 300 °C are 2.1%, 16.9%, and 20.8%, respectively, following a pattern characterized by a mild decline at lower temperatures and an accelerated decline at higher temperatures. At 100 °C, only free water evaporation took place within the concrete, leaving the cement paste structure largely undamaged and thus resulting in a negligible strength loss. At 200 °C, dehydration of the C-S-H gel occurred, impairing the cementitious binding capacity and elevating the reduction to 16.9%. At 300 °C, the cement paste structure became further loosened, with the reduction reaching 20.8%. This stage-dependent behavior aligns well with established theories regarding the high-temperature mechanical response of concrete, thereby corroborating the reasonableness of the thermal effects examined in this study. For the RC5 specimens, the corresponding reductions at 100 °C, 200 °C, and 300 °C are 6.3%, 12.7%, and 18.4%, respectively, which likewise exhibited a mild-to-accelerated pattern. Nevertheless, the degradation behavior across temperature intervals deviated from that of the NC group. At 100 °C, the elastic buffering effect of rubber offset part of the strength loss induced by free water evaporation; however, the reduction for RC5 still surpassed that of NC. This is because the rubber–cement interface is more susceptible to moisture migration. In the 200–300 °C range, despite the interfacial damage caused by rubber thermal degradation, the plastic deformation of rubber particles continued to dissipate a portion of the energy, resulting in an overall strength reduction smaller than that of NC. This observation highlights the buffering role of rubber particles against high-temperature deterioration. For the RC15 specimens, the strength reductions at 100 °C, 200 °C, and 300 °C are 7.7%, 15.3%, and 24.9%, respectively—the largest among the three groups at elevated temperatures. At 100 °C, the strength reduction exceeded those of both NC and RC5, owing to the heightened sensitivity of interfacial defects to moisture transport. In the 200–300 °C interval, extensive thermal degradation of rubber generated a substantial increase in voids and microcracks, driving the strength reduction far beyond those observed for NC and RC5. This finding further substantiates the combined detrimental effect of high rubber content and elevated temperature.

### 4.3. Dynamic Failure Phenomenon

**Figure 8:**
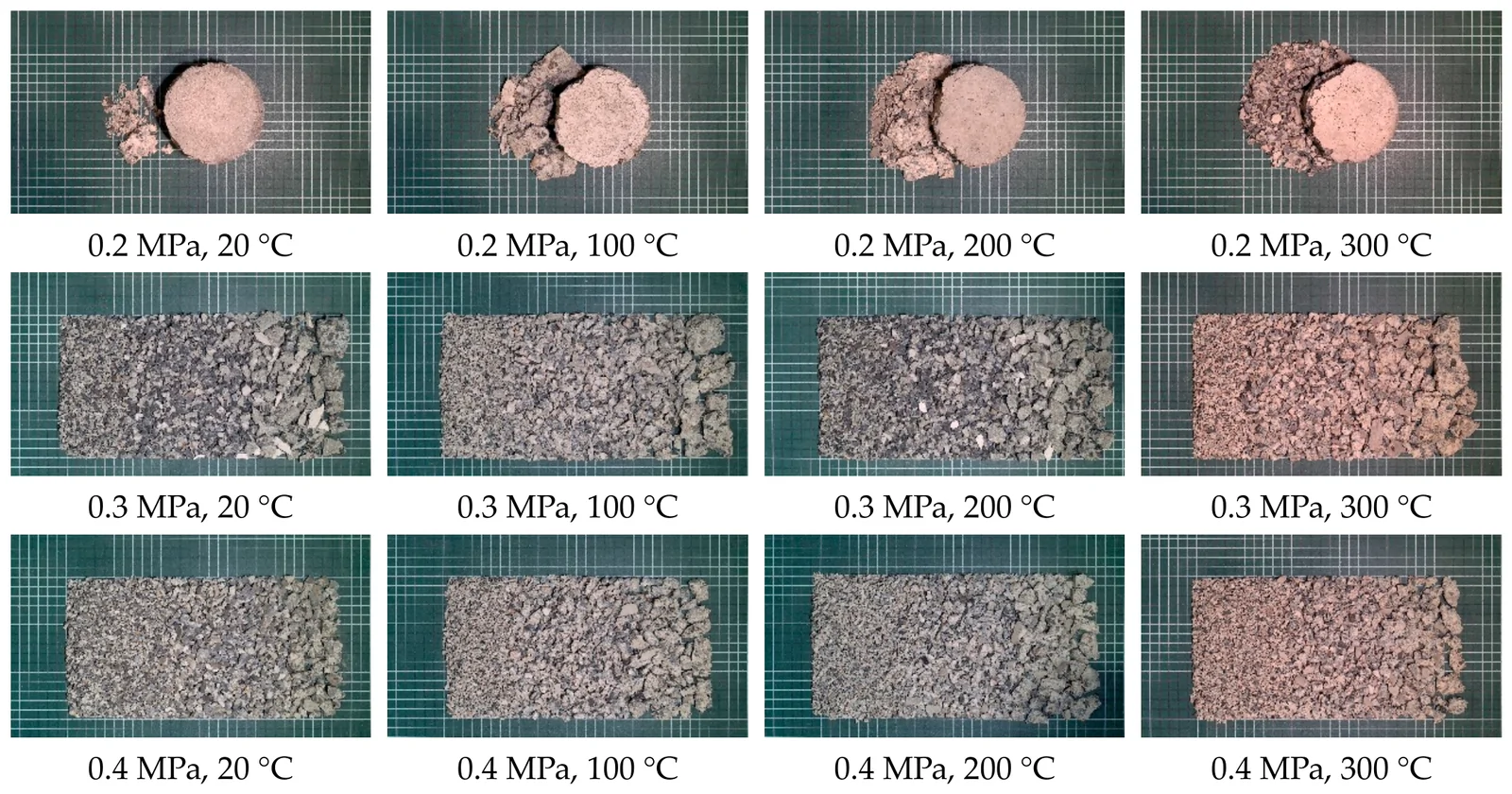
Fracture morphology of NC.

**Figure 9:**
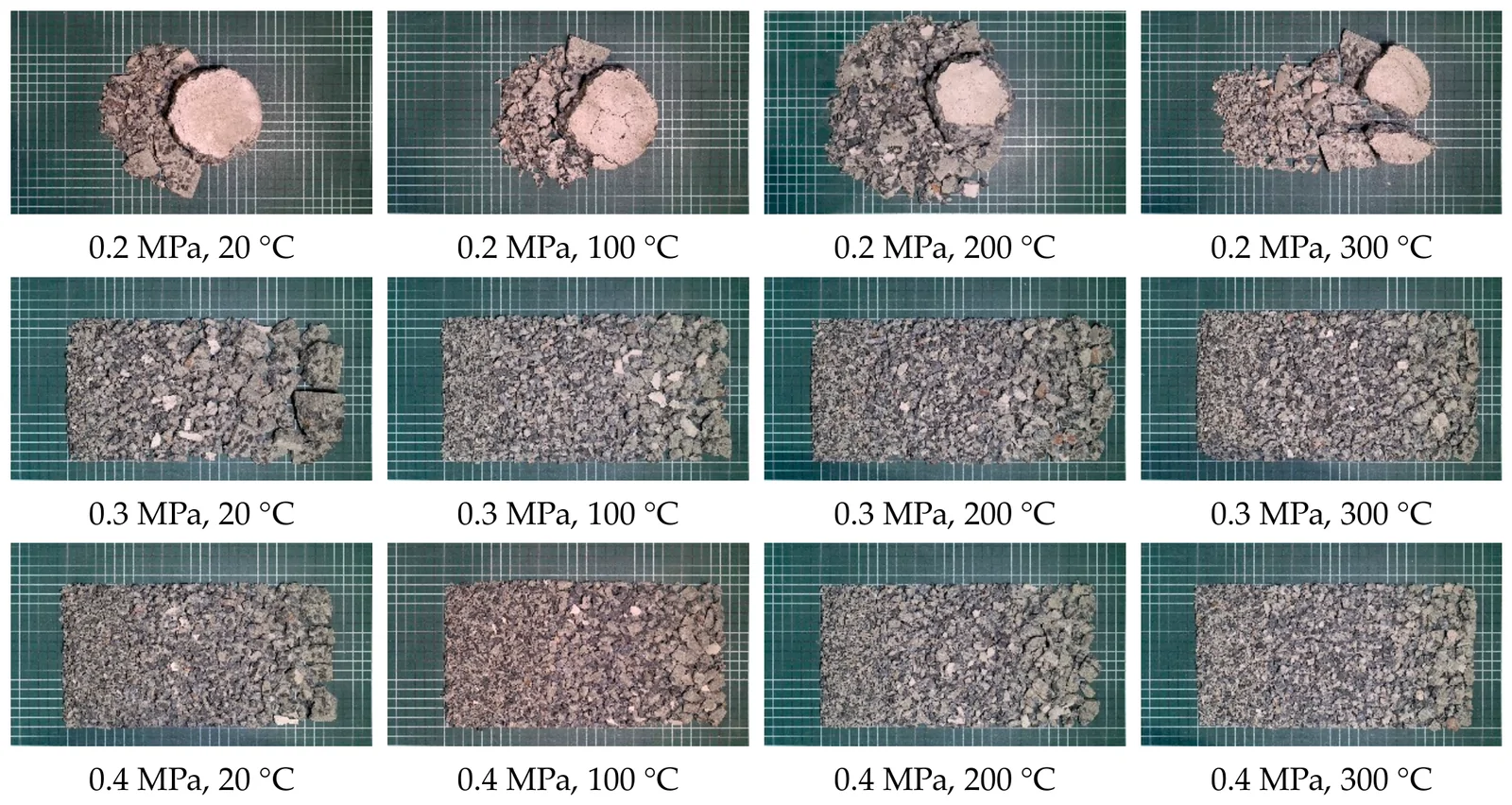
Fracture morphology of RC5.

**Figure 10:**
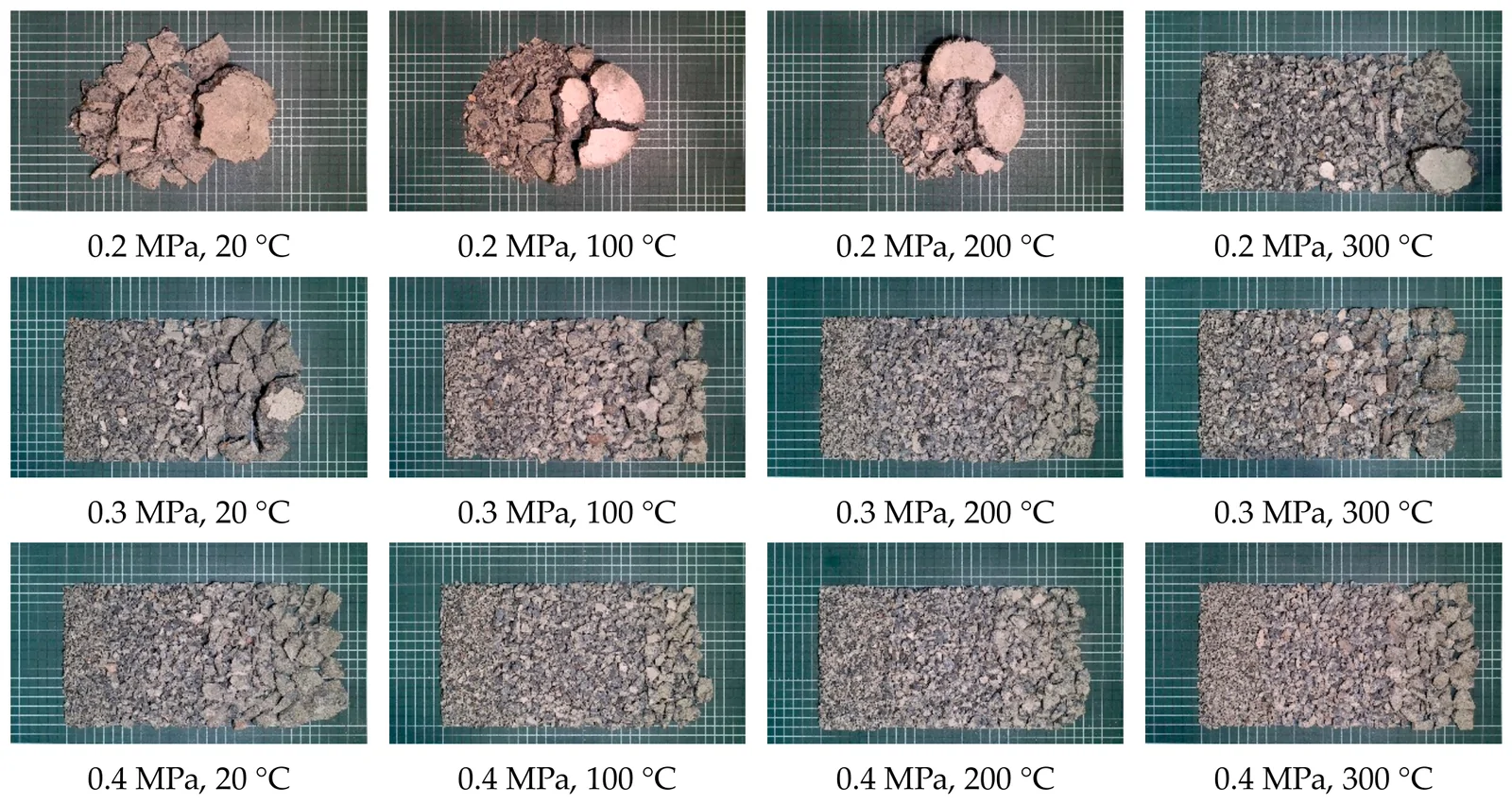
Fracture morphology of RC15.

The failure mode of concrete specimens subjected to impact loading provides direct insight into the mechanisms of crack initiation, propagation, and coalescence under dynamic stress states, thus constituting a critical basis for evaluating dynamic mechanical behavior [31]. In the present study, the failure mode of the specimens is governed by three primary factors—impact air pressure (i.e., strain rate), rubber content, and exposure temperature—each exerting a distinctly different level of influence. The fragmentation patterns of the specimens following dynamic impact are presented in Figure 8, Figure 9 and Figure 10.

Rubber content modulates the dynamic fragmentation behavior by altering the meso-structure and interfacial characteristics of the concrete; however, this effect is pronounced only under the low air pressure condition of 0.2 MPa. For the RC5 specimens, the rubber particles are dispersed relatively uniformly within the cementitious matrix, with no apparent weak-bonding features at the ITZ, and the matrix retained a high degree of structural continuity. As a result, under low-velocity impact, stress concentration is confined to localized rubber–matrix interfaces, inducing only limited crack propagation, while the primary structure of the specimens remained largely intact. Accordingly, the degree of fragmentation is substantially lower than that observed for the RC15 specimens under identical conditions.

For the RC15 specimens, the higher rubber content pronouncedly increased the number of weak internal interfaces within the matrix. In addition, the low elastic modulus of the rubber phase further aggravated the stress concentration effect. Concurrent debonding and cracking occurred at multiple rubber–matrix interfaces, which markedly promoted the efficiency of crack initiation and propagation. Experimental observations revealed that under the conditions of 0.2 MPa and 20 °C, the fragment proportion of the RC15 group is considerably higher than that of the RC5 group. Under 0.2 MPa and 300 °C, the RC15 specimens are almost completely comminuted, with only fragments smaller than 10 mm remaining. This finding further corroborates the facilitating role of high rubber content in specimen fragmentation under low-velocity impact.

Beyond rubber content, both impact air pressure and temperature exert notable influences on the fragmentation characteristics of the specimens, with a distinct hierarchy among these three factors. Impact air pressure directly governs the energy intensity of the incident wave in the SHPB tests and thus constitutes the primary determinant of the extent of specimen fragmentation. At an air pressure of 0.2 MPa, none of the specimens underwent complete failure; instead, they exhibited localized crushing or radial cracking. Specifically, the NC specimens retained relatively large macroscopic fragments after testing, with only minor spalling at the edges. The RC5 specimens preserved a main block structure exceeding 20 mm in diameter, accompanied by only a few small fragments smaller than 5 mm. Although the RC15 specimens show a slightly higher fragment proportion, macroscopic residual block structures are still observable. These observations indicate that the impact energy delivered at 0.2 MPa fell below the dynamic impact strength threshold of RC, thus inducing only localized crack propagation without achieving global matrix disintegration.

When the impact air pressure is raised to 0.3 MPa and 0.4 MPa, all specimens—irrespective of rubber content—exhibited global crushing failure characterized by full-gradation granulation, with no macroscopic block remnants larger than 20 mm in diameter; the fragmentation products are predominantly particles ranging from 2 mm to 10 mm in size. This transition is attributable to the substantial increase in incident wave energy under elevated air pressure, which caused the impact load to surpass the dynamic peak strength of RC. Consequently, multiple cracks initiated, coalesced, and propagated unstably throughout the matrix, culminating in complete specimen fragmentation. Under these high-pressure conditions, impact energy played an overwhelmingly dominant role, overwhelming any modulating effects of rubber content or temperature—specimens failed completely regardless of whether they were tested at ambient temperature or 300 °C.

The influence of temperature on fragmentation characteristics exhibited a clear rubber-content dependence, and this synergistic effect is observable only under the low air pressure condition of 0.2 MPa; at higher air pressures, its contribution is entirely eclipsed by the dominant impact energy. At 0.2 MPa, as the temperature rose from 20 °C to 300 °C, the degree of fragmentation increased progressively, as evidenced by a reduction in macroscopic fragments and a corresponding increase in fine particles. This trend suggests that elevated temperatures lower the dynamic fragmentation energy threshold through multiple mechanisms—including reduced dynamic strength, deterioration of the interfacial transition zone, and promotion of internal microcrack propagation—thereby aggravating the extent of fragmentation.

In greater detail, for the RC15 specimens, high temperature intensified the degradation of the rubber phase and exacerbated meso-structural defects within the matrix. Under low-velocity impact, the thermally softened rubber particles are susceptible to plastic deformation, while the bond strength at weak interfaces is further compromised, substantially reducing crack propagation resistance. As a result, under 0.2 MPa and 300 °C, the fragmentation degree of the RC15 group is considerably more severe than that at 20 °C under the same air pressure. For the RC5 specimens, owing to their lower rubber content, the thermal softening effect exerted only a marginal influence on the overall matrix performance; consequently, over the 20–300 °C range, the fragmentation characteristics exhibited only a slight increase in fragment count, without any marked enhancement in fragmentation severity. The NC specimens are the least sensitive to temperature, showing only a minor increase in fine particles at 300 °C, while the overall fragmentation degree remained essentially unchanged—a trend consistent with the stable static mechanical performance of NC following high-temperature exposure. In summary, the dynamic fragmentation behavior of RC exhibits a well-defined hierarchy of influencing factors: impact air pressure is the primary controlling factor, dictating whether the failure mode is localized or complete; rubber content serves as the key modulating factor under low-velocity impact, influencing the extent of fragmentation by altering the meso-structure; and the temperature–rubber content interaction enhances fragmentation only under low-velocity, high-content conditions, an effect that is completely masked by the dominance of impact energy at elevated air pressures.

### 4.4. Dynamic Stress–Strain Characteristics

**Figure 11:**
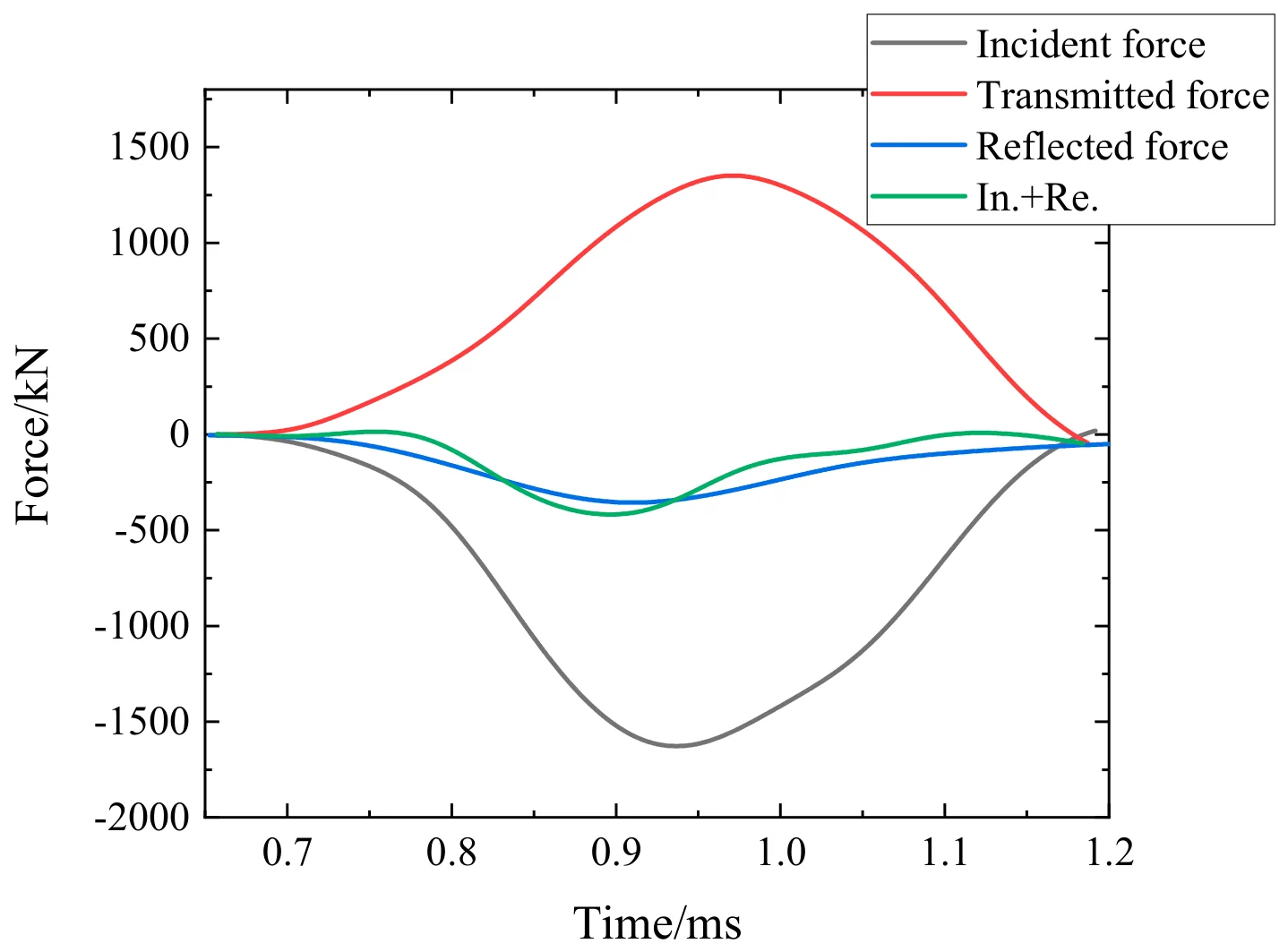
Dynamic force equilibrium of RC5 specimens after 300 °C exposure at a strain rate of ~100 s^−1^.

The dynamic force balance of the specimen can be used to verify the accuracy of the test results, as shown in Figure 11. For brevity, only one representative condition (among the numerous combinations of temperature, rubber content, and impact pressure) is presented. Figure 11 demonstrates excellent force balance on both sides of the specimen, confirming the validity of the dynamic tests; analogous equilibrium states are confirmed for all other test conditions. Using the two-wave method based on the assumption of stress uniformity ($\varepsilon_{i}(t)+\varepsilon_{r}(t)=\varepsilon_{t}(t))$, the compression strain rate $\dot{\varepsilon_{s}}(t)$, the compressive strain $\varepsilon_{s}(t)$ and the compressive stress $\sigma_{s}(t)$ can be calculated using the following Equation (2).

(2) $$\{\begin{array}{c} \dot{\varepsilon_{s}}(t)=-\frac{2c_{b}}{l_{0}}\varepsilon_{r}(t)\\ \varepsilon_{s}(t)=-\frac{2c_{b}}{l_{0}}\int_{0}^{t}\varepsilon_{r}(\tau )\\ \sigma_{s}(t)=\frac{r_{b}^{2}}{r_{0}^{2}}E_{b}\varepsilon_{t}(t) \end{array}$$

$E_{b}$ is the Young’s modulus of the elastic bar, 206,000 MPa. $c_{b}$ is the stress wave velocity in the bar, 5190 m/s. $r_{b}$ and $r_{0}$ are the radius of the bar and the sample. $l_{0}$ is the length of the sample. $\varepsilon_{i}$, $\varepsilon_{r}$ and $\varepsilon_{t}$ are incident, reflected, and Transmitted strains in the bar, respectively.

**Figure 12:**
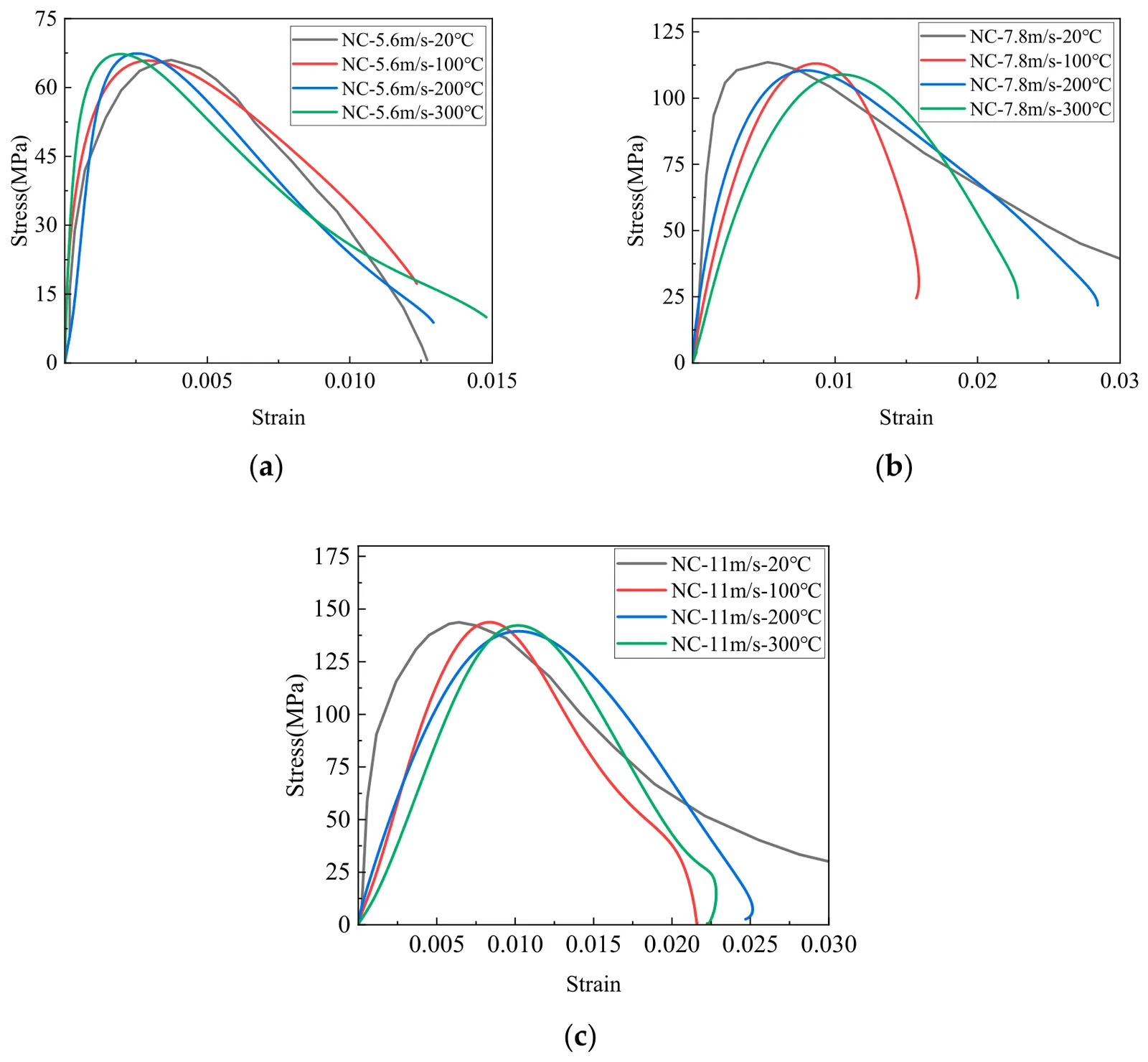
Dynamic stress–strain curve of NC under different strain rates. (**a**) 100 s^−1^; (**b**) 140 s^−1^; (**c**) 170 s^−1^.

Figure 12 presents the complete dynamic stress–strain curves of the NC specimens under various strain rates and temperatures. As can be seen, the dynamic stress–strain response of NC exhibits pronounced strain-rate sensitivity, whereas temperature exerts a negligible influence on both the curve morphology and the characteristic parameters.

With regard to strain-rate effects, the dynamic peak stress of the NC specimens increased continuously and substantially with rising impact air pressure and strain rate. As the strain rate elevated from approximately 100 s^−1^ to roughly 170 s^−1^, the peak stress rose from 66 MPa to 143.7 MPa—an increase of 117.7%—demonstrating a clear strain-rate strengthening effect on the dynamic strength of NC. Under identical strain-rate conditions, no notable changes are observed in the curve shape, peak stress, ascending-branch slope, or descending-branch features of the NC specimens across the entire 20–300 °C temperature range. The maximum fluctuation in peak stress over this temperature interval did not exceed 4.1%. The above results serve as a benchmark for the subsequent analysis of the dynamic mechanical behavior of RC, enabling a clear comparison of the fundamental differences between RC and NC in terms of dynamic stress response, strength evolution, and failure characteristics.

**Figure 13:**
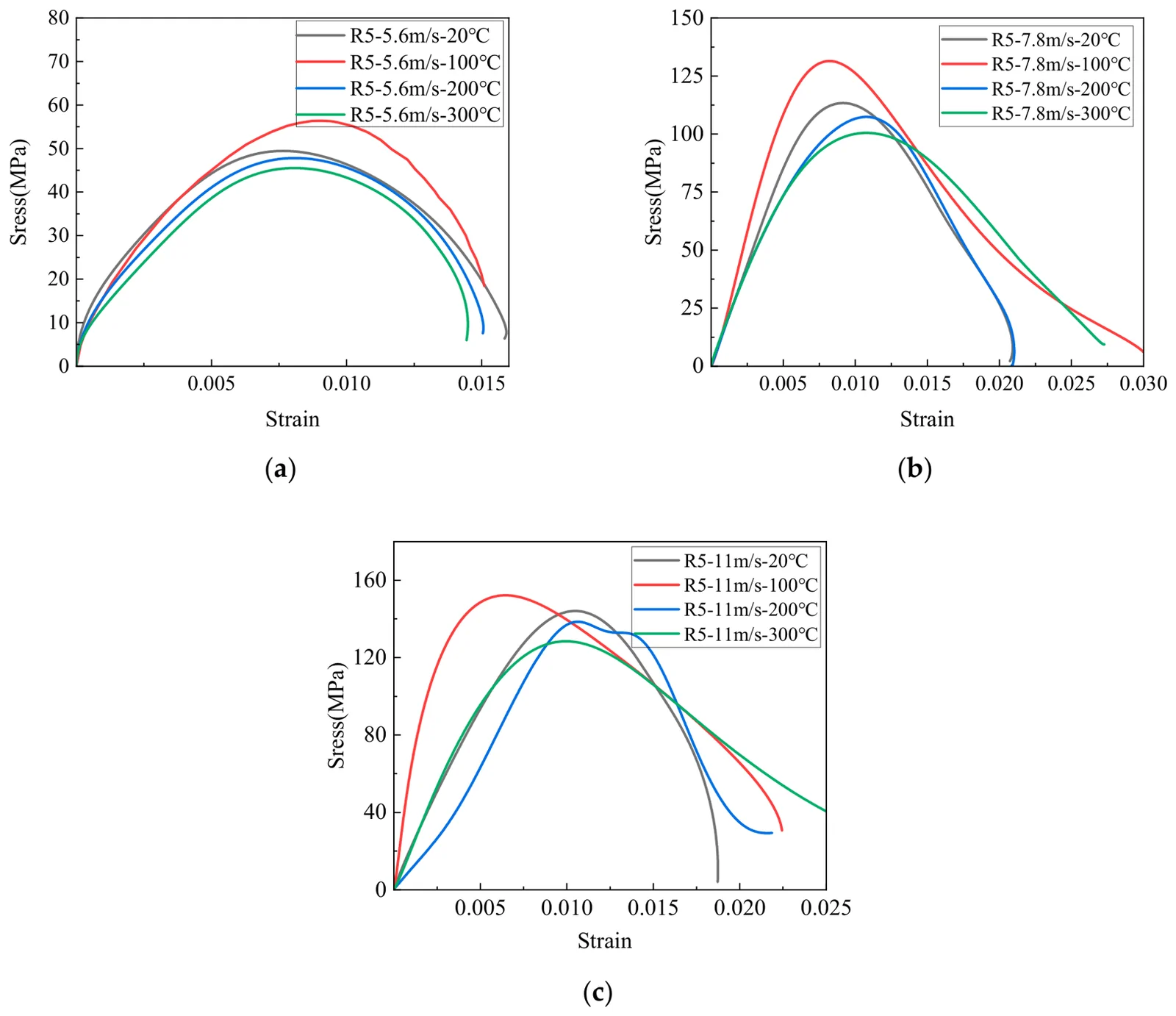
Dynamic stress–strain curve of RC5. (**a**) 100 s^−1^; (**b**) 140 s^−1^; (**c**) 170 s^−1^.

Figure 13 presents the complete dynamic stress–strain curves of the RC5 specimens under different strain rates and temperatures. The results indicate that the dynamic mechanical properties of the RC5 specimens are markedly sensitive to both temperature and strain rate. The dynamic strength exhibits a two-stage evolution with increasing temperature—an initial pronounced enhancement followed by progressive degradation—and this pattern remains consistent across the entire strain-rate spectrum.

At a low strain rate (~100 s^−1^, 0.2 MPa), the peak stress recorded at 20 °C is 49.5 MPa; it surged to 56.4 MPa at 100 °C (a 14.6% increase), dropped to 47.8 MPa at 200 °C, and further declined to 45.5 MPa at 300 °C. At a medium strain rate (~140 s^−1^, 0.3 MPa), the peak stress is 113.4 MPa at 20 °C; it climbed to 131.5 MPa at 100 °C (a 15.9% increase), fell to 107.4 MPa at 200 °C, and decreased to 100.5 MPa at 300 °C. At a high strain rate (~170 s^−1^, 0.4 MPa), the peak stress is 144.1 MPa at 20 °C; it rose to 152.2 MPa at 100 °C (a 5.6% increase), dropped to 138.5 MPa at 200 °C, and fell to 128.5 MPa at 300 °C.

In terms of curve deformation characteristics, under the 100 °C condition, the stress–strain curves of the RC5 specimens exhibit higher peak stresses, larger peak strains, and a more gradual descending branch, indicating favorable dynamic ductility. Under the 200 °C and 300 °C conditions, the descending branch becomes steeper, the peak strain diminishes, and ductility is markedly reduced. These findings suggest that RC5 achieves its optimal dynamic mechanical performance at 100 °C, combining relatively high dynamic strength with good deformability. At temperatures of 200 °C and above, both dynamic strength and ductility undergo considerable deterioration, underscoring the adverse impact of elevated temperatures on dynamic mechanical behavior.

**Figure 14:**
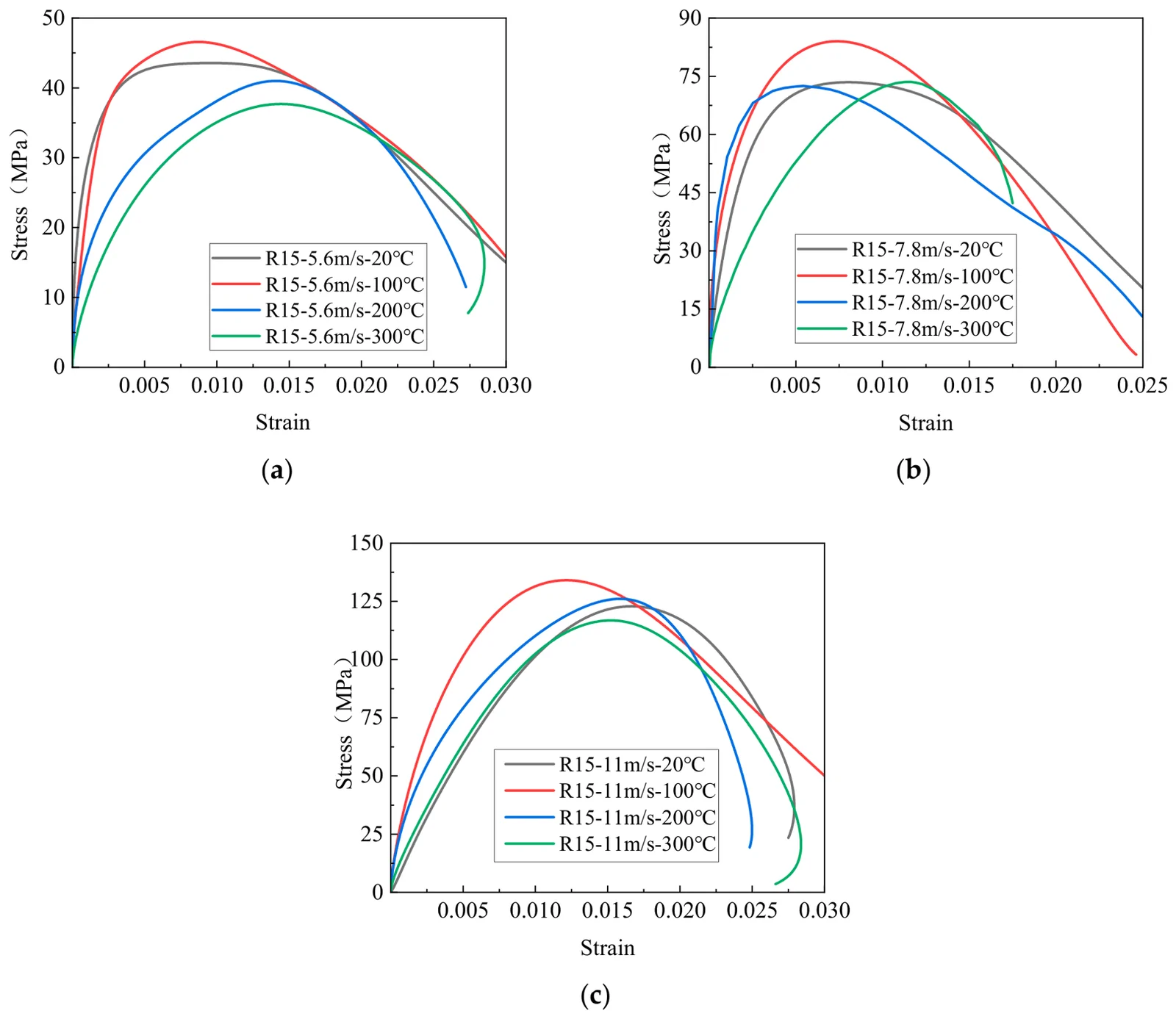
Dynamic stress–strain curve of RC15. (**a**) 100 s^−1^; (**b**) 140 s^−1^; (**c**) 170 s^−1^.

Figure 14 presents the complete dynamic stress–strain curves of the RC15 specimens under different strain rates and temperatures. As can be observed, the temperature-dependent trend of dynamic strength for the RC15 specimens is consistent with that of the RC5 group—also exhibiting notable strengthening at 100 °C and gradual degradation at 200 °C and above. However, the strength retention at elevated temperatures is superior, and the overall fluctuation amplitude narrows as the strain rate increases.

At a low strain rate (~100 s^−1^, 0.2 MPa), the peak stress at 20 °C is 43.6 MPa; it rose to 46.5 MPa at 100 °C (a 6.6% increase), fell to 41.0 MPa at 200 °C, and dropped to 37.7 MPa at 300 °C—a reduction of 13.5% relative to the 20 °C value. At a medium strain rate (~140 s^−1^, 0.3 MPa), the peak stress is 73.5 MPa at 20 °C; it climbed to 84.0 MPa at 100 °C (a 14.3% increase), dropped to 72.5 MPa at 200 °C, and recovered slightly to 73.5 MPa at 300 °C, with the maximum peak-stress variation across the entire temperature range not exceeding 10.5 MPa. At a high strain rate (~170 s^−1^, 0.4 MPa), the peak stress is 122.9 MPa at 20 °C; it rose to 134.1 MPa at 100 °C (a 9.1% increase), fell to 126.1 MPa at 200 °C, and decreased to 116.8 MPa at 300 °C, with a reduction of 12.9% relative to the peak value.

In terms of curve deformation characteristics, the descending branch of the stress–strain curves for the RC15 specimens are considerably gentler than that of the RC5 and NC groups under identical conditions, accompanied by larger peak strains, reflecting superior deformability and toughness. Even at 300 °C, the descending branch retains a gradual shape without exhibiting a steep stress drop, indicating that high-content rubber particles can still impede crack propagation through plastic deformation at elevated temperatures, thereby preserving the ductility of the material.

The energy dissipation characteristics of concrete under impact loading constitute a critical metric for assessing impact resistance and protective performance, as they directly reflect the material’s capacity to absorb impact energy through mechanisms such as plastic deformation, interfacial debonding, and crack propagation during the impact event [32]. In the following subsection, based on the complete dynamic stress–strain curves, the evolution patterns of total energy dissipation and energy dissipation efficiency of RC under impact loading are systematically examined, with the objective of elucidating the coupled effects of strain rate, temperature, and rubber content on the energy dissipation behavior.

### 4.5. Strain-Rate

The strain-rate effect describes the variation in mechanical properties of concrete materials with changing loading strain rates. To quantitatively assess the intensity of this effect, the DIF is introduced, defined as the ratio of the dynamic peak strength at a given strain rate to the quasi-static compressive strength at the same temperature, as expressed in Equation (3):

(3) $$\mathrm{DIF}=\frac{f_{d,\varepsilon }}{f_{s,T}}$$

where $f_{d,\varepsilon }$ is the dynamic peak strength (MPa) at a given strain rate *ε*, obtained directly from experimental measurements; $f_{s,T}$ is the static compressive strength (MPa) at the same temperature *T*.

Using the measured data from the SHPB tests and the static compressive strength results, the DIF values of specimens under various conditions are calculated, with detailed results listed in Table 5, Table 6 and Table 7. It is evident that the DIF of all specimen groups increases with rising strain rate. Higher rubber content is associated with greater DIF sensitivity to strain rate, reflecting a more pronounced strain-rate strengthening effect. Moreover, as temperature and rubber content increase, the scatter in DIF gradually becomes more pronounced. This phenomenon is attributable to the coupled influence of elevated temperature and high rubber content, which introduces specimen-to-specimen variations in the degree of thermally induced internal deterioration, thereby amplifying the dispersion of the strain-rate strengthening effect. It should be noted that the DIF in this study is calculated as the ratio of quasi-static cubic strength to dynamic cylindrical/disk strength. Since there is currently no universally accepted conversion coefficient between different specimen geometries under dynamic loading conditions, the DIF values reported herein are primarily applicable for relative comparisons within the same material system.

### 4.6. Energy Characteristics

#### 4.6.1. Energy Dissipation

The total energy dissipation ($W_{t}$) of specimens under dynamic impact is characterized by the area enclosed by the complete dynamic stress–strain curve, representing the total energy absorbed per unit volume from the onset of loading to complete failure. It is calculated as Equation (4):

(4) $$W_{t}=\int_{0}^{\varepsilon_{f}}\sigma_{D}\left(\varepsilon \right)d\varepsilon$$

where $W_{t}$ is the total energy dissipation of the specimen under impact loading (kJ/m^3^), $\sigma_{D}\left(\varepsilon \right)$ is the dynamic stress, and $\varepsilon_{f}$ is the dynamic failure strain.

As shown in Table 8, the calculated total energy dissipation under various test conditions demonstrates that strain rate is the dominant factor governing the magnitude of energy absorption. The total energy dissipation increased substantially with rising impact air pressure and strain rate. At strain rates of approximately 100 s^−1^, the total energy dissipation ranged from 161.4 to 369.2 kJ/m^3^; at around 140 s^−1^, it increased to 327.0–915.7 kJ/m^3^; and at approximately 170 s^−1^, it further rose to 604.8–1078.0 kJ/m^3^. Notably, the energy dissipation of NC at 140 s^−1^ and 20 °C reached 915.7 kJ/m^3^, which is substantially higher than the values observed under other conditions. Overall, as the strain rate increased from roughly 90 s^−1^ to 180 s^−1^, the total energy dissipation of all specimens exhibited pronounced growth. This trend indicates that the elastic deformation of rubber particles, interfacial friction, and fracture energy dissipation of the concrete matrix are all remarkably enhanced under high strain-rate loading. However, the influence of temperature on energy dissipation is less consistent than that of strain rate, with no clear monotonic trend across the investigated temperature range. This suggests that strain rate plays a more dominant role than temperature under the present experimental conditions. It is also worth noting that, while RC15 exhibited relatively high energy dissipation values under certain high-strain-rate and elevated-temperature conditions (e.g., 780.9 kJ/m^3^ at 174.9 s^−1^ and 300 °C), the energy dissipation of NC under certain conditions (e.g., 915.7 kJ/m^3^ at 140 s^−1^ and 20 °C) is even higher. Thus, rather than rubber content being a single determining factor, the results suggest that the interplay among strain rate, temperature, and rubber content collectively governs the energy dissipation capacity.

#### 4.6.2. Pre-Peak Energy Fraction

To quantitatively characterize the utilization efficiency of energy conversion and transfer within the system, the pre-peak energy fraction ($\eta_{p}$) is introduced as an evaluation index. It is mathematically defined as Equation (5):

(5) $$\eta_{p}=\frac{\int_{0}^{\varepsilon_{p}}\sigma_{D}\left(\varepsilon \right)\mathrm{d}\varepsilon }{W_{t}}\times 100\%$$

where $\eta_{p}$ is the pre-peak energy fraction, and $\varepsilon_{p}$ is the peak strain.

**Figure 15:**
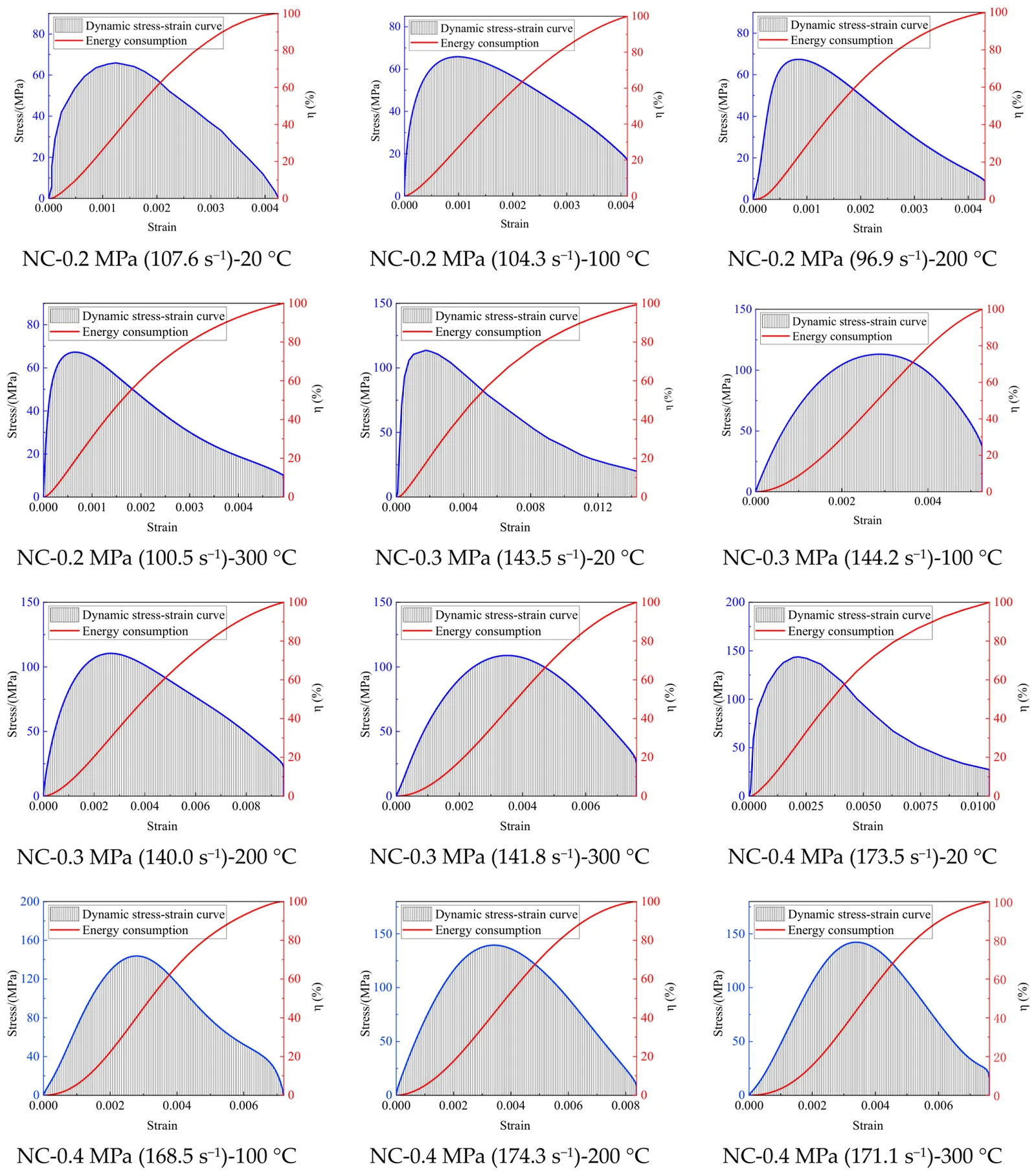
Energy dissipation efficiency of NC.

If $\eta_{p}>50\%$, more energy is dissipated before failure than after, with energy primarily consumed in the elastic and plastic deformation stages. If $\eta_{p}<50\%$, more energy is dissipated after failure than before, with the majority of energy expended in post-peak crack propagation and matrix fragmentation. As shown in Figure 15, the total energy dissipation of the NC group exhibited a stable, upward trend with increasing air pressure, while temperature exerted a negligible influence. At 0.2 MPa, the total energy dissipation remained within 200–240 kJ/m^3^, with virtually no variation across different temperatures; at 0.3 MPa, it increased to 350–420 kJ/m^3^; and at 0.4 MPa, it further rose to 620–680 kJ/m^3^, with each strain-rate gradient yielding a stable increment of approximately 70%. The evolution of energy dissipation efficiency displayed a clear strain-rate dependence: under low strain rates, $\eta_{p}$ exceeded 50%, with energy dissipation concentrated in the elastic and plastic deformation stages preceding the peak strength; under high strain rates, $\eta_{p}$ dropped below 40%, with most energy dissipated in post-peak crack propagation and matrix fragmentation. Meanwhile, the asymmetry of the dynamic stress–strain curves intensified with increasing strain rate, whereas rising temperature progressively improved curve symmetry and substantially reduced the stiffness disparity before and after the peak.

**Figure 16:**
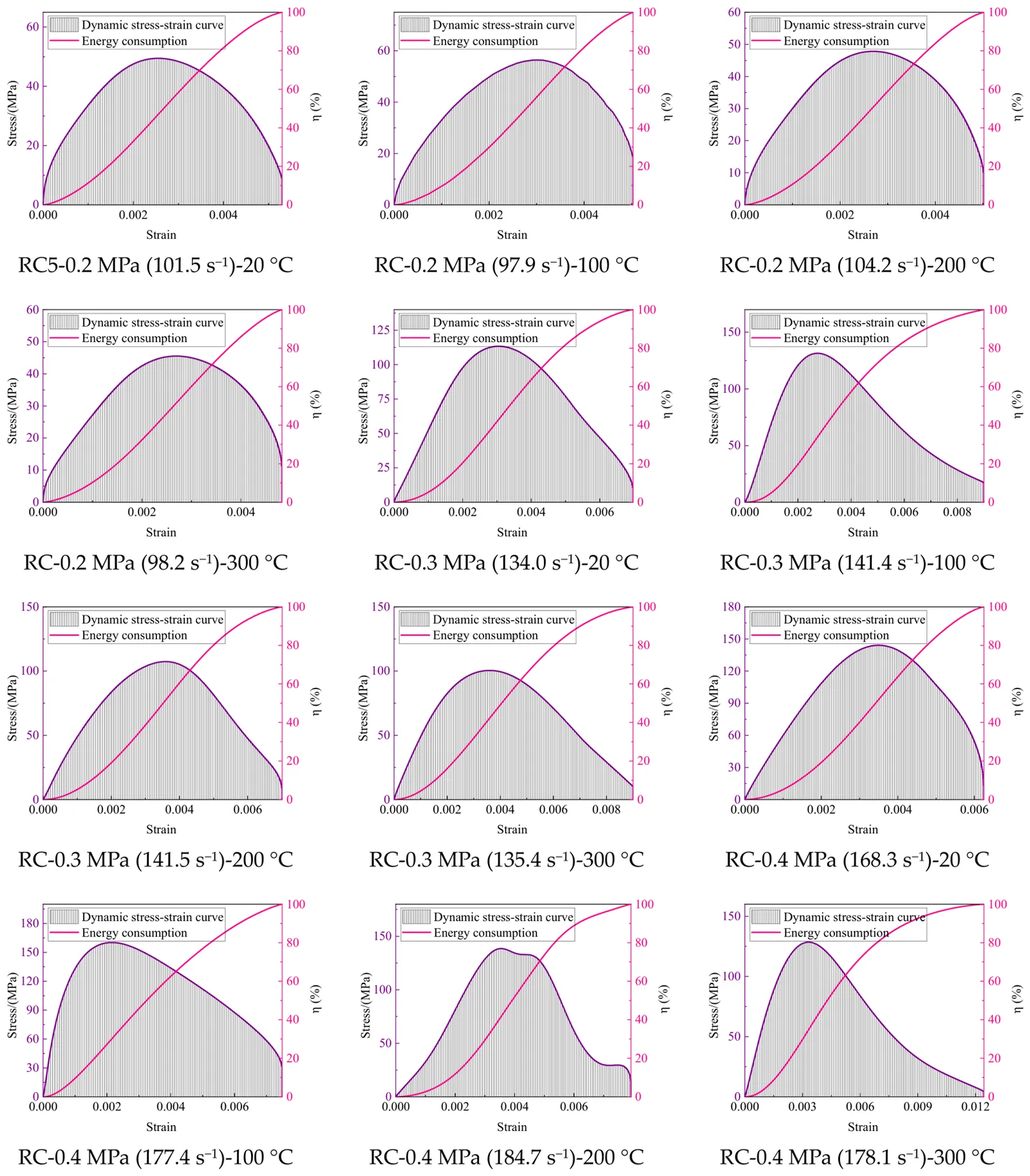
Energy dissipation efficiency of RC5.

As shown in Figure 16, the increase in total energy dissipation of the RC5 group with rising strain rate far exceeded that of NC, while also exhibiting a pronounced temperature dependence characterized by a single-peak pattern of initial increase followed by decline. At 0.2 MPa, the total energy dissipation ranged from 161.4 to 320 kJ/m^3^—comparable to NC at 20 °C, surging to 320 kJ/m^3^ at 100 °C (nearly double that at 20 °C), and gradually decreasing at 200 °C and 300 °C, reaching the group’s minimum of 161.4 kJ/m^3^ at 300 °C. At 0.3 MPa, the total energy dissipation increased to 400–720 kJ/m^3^, with 100 °C remaining the peak point (a 45% increase over 20 °C). At 0.4 MPa, the total energy dissipation rose to 750–980 kJ/m^3^, with the fluctuation amplitude across the temperature range further narrowing, indicating that the dominant effect of strain rate gradually overshadowed the modulating influence of temperature. The energy dissipation efficiency exhibited a clear single-peak characteristic with temperature: under low strain rates, $\eta_{p}$ remained within 55–65%, with peak efficiency at 100 °C; under high strain rates, $\eta_{p}$ dropped to below 45%, with plastic deformation of rubber particles and interfacial friction becoming the primary dissipation mechanisms, and the ascending slope of the efficiency curve accelerated markedly with increasing strain rate, approaching saturation more rapidly.

**Figure 17:**
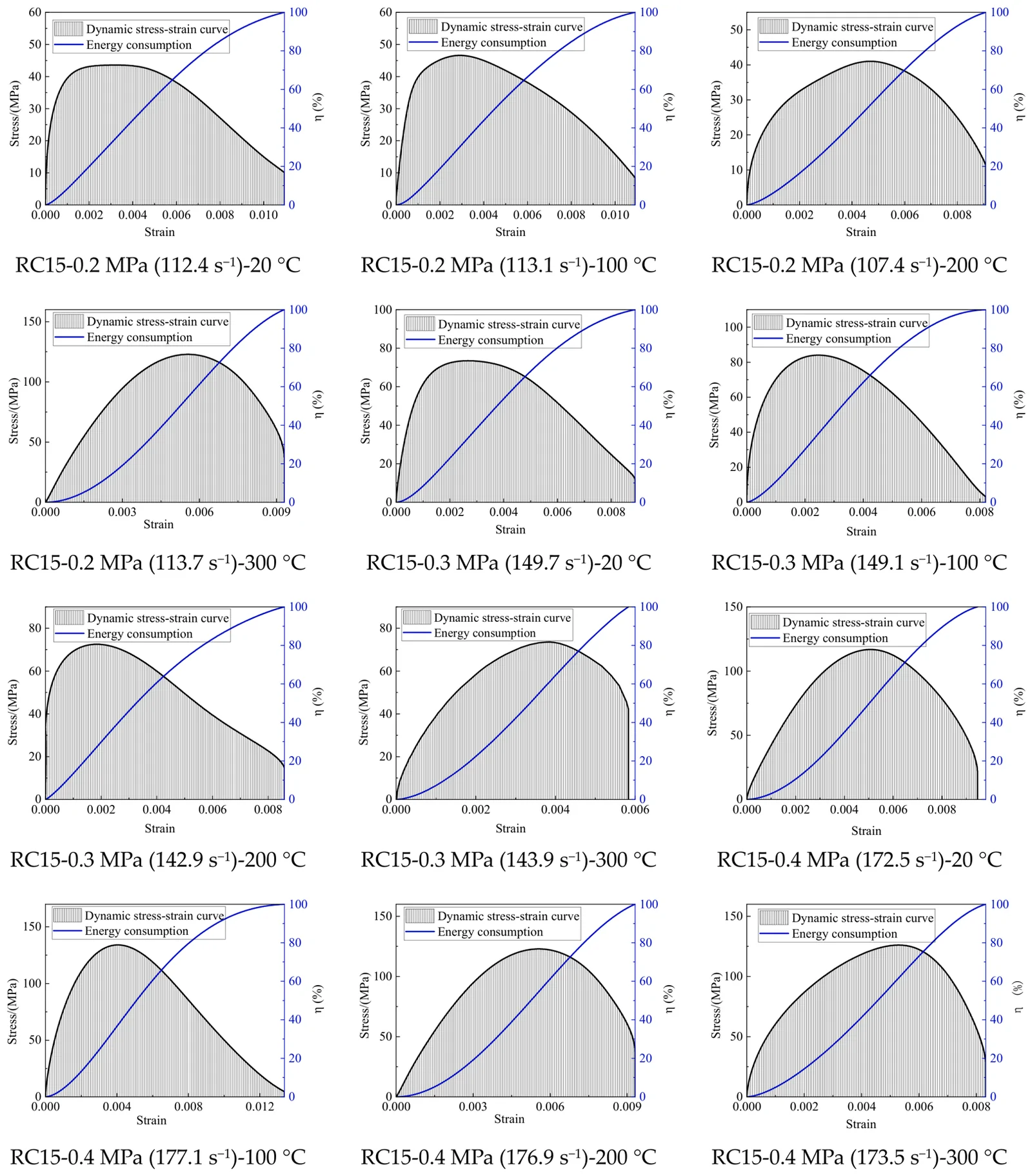
Energy dissipation efficiency of RC15.

As shown in Figure 17, the total energy dissipation of the RC15 group is the highest among the three groups across the entire strain-rate range, while also exhibiting the most pronounced temperature synergistic effect and strain-rate strengthening effect, with energy dissipation retention at elevated temperatures far superior to that of the RC5 group. At 0.2 MPa, the total energy dissipation ranged from 280 to 369.2 kJ/m^3^—even at 20 °C, it exceeded that of NC under the same air pressure by more than 50%, reaching the group’s maximum of 369.2 kJ/m^3^ at 100 °C, and although it decreased at 200 °C and 300 °C, it remained higher than NC and RC5. At 0.3 MPa, the total energy dissipation increased to 600–915.7 kJ/m^3^, with the 100 °C peak showing a 52% increase over 20 °C, and still maintaining above 780 kJ/m^3^ at 300 °C. At 0.4 MPa, the total energy dissipation rose to 850–1078 kJ/m^3^, reaching the highest value of 1078 kJ/m^3^ at 100 °C across all test conditions, and even at 300 °C, it remained above 920 kJ/m^3^, demonstrating exceptional stability across the entire temperature range.

The energy dissipation efficiency of the RC15 group is the optimal among the three groups under all test conditions, exhibiting the highest energy utilization efficiency and stability. Under low strain rates, $\eta_{p}$ remained within 60–70%, with the high plastic deformability of rubber particles enabling most energy to be effectively absorbed before the peak. Under high strain rates, $\eta_{p}$ could still be maintained at approximately 48%, much higher than that of NC and RC5 at equivalent strain rates; after the peak, impact energy could still be continuously dissipated through diffuse multi-cracking failure, with the descending branch of the efficiency curve being the gentlest, devoid of any rapid drop.

From the perspective of post-peak energy dissipation, the $\eta_{p}$ values of all specimens are less than 50% under high strain rates. This is attributable to the fact that under high strain-rate conditions, a single critical crack does not form within the specimen; instead, multiple cracks initiate and develop simultaneously. The entire process—from compaction and densification through crack initiation to propagation—is completed within an extremely short timeframe, consuming a substantial amount of impact energy, which consequently results in a higher proportion of post-peak energy dissipation.

## 5. Conclusions

This study investigated the residual performance evolution mechanisms of RC after exposure to elevated temperatures and subsequent cooling to room temperature. A comprehensive experimental program comprising static compressive tests, splitting tensile tests, and SHPB dynamic impact tests was conducted. The main findings are summarized as follows:

(1) Both temperature and rubber content affect the apparent characteristics and mass loss of RC after high-temperature exposure. Apparent quality progressively deteriorated with increasing temperature; at 300 °C, specimens turned reddish-brown with increased cracking and reduced compactness. The mass loss rate increased monotonically with temperature, with higher rubber content leading to greater loss. At 300 °C, the 30% rubber content specimens reached a mass loss rate of 7.99% attributable to the synergistic effects of moisture evaporation, hydration product decomposition, and rubber thermal degradation.

(2) The coupled effect of temperature and rubber content substantially influences the residual static mechanical properties of RC. Compressive strength decreased monotonically with rising temperature, with higher rubber content leading to more pronounced degradation. The compressive strength of RC30 dropped by approximately 40.6% from ambient temperature to 300 °C, while at 300 °C, the strength of RC30 is 64.6% lower than that of NC.

(3) Both DIF and energy dissipation increase with strain rate, and the strain-rate sensitivity of DIF is influenced by rubber content and temperature. Overall, higher rubber content tends to enhance the strain-rate sensitivity of DIF under elevated-temperature conditions. At a strain rate of approximately 100 s^−1^, rubberized mixtures generally exhibit higher energy dissipation than NC, with energy dissipation increasing with rubber content. However, at strain rates of approximately 140 s^−1^ and 170 s^−1^, the relationship between rubber content and energy dissipation becomes less clear, with no consistent monotonic trend observed across the different rubber contents and temperatures.

## Figures and Tables

**Table 1:** Mix proportions of concrete.

	Water (kg/m^3^)	Cement (kg/m^3^)	Coarse Aggregate (kg/m^3^)	Fine Aggregate (kg/m^3^)	Rubber Particles (kg/m^3^)
NC	220	489	1113	523	0
RC5	220	489	1113	487	11
RC15	220	489	1113	445	34
RC30	220	489	1113	336	68

**Table 2:** Parameters related to dynamic tests.

	Air Pressure (MPa)	Velocity (m/s)	Pulse Shaper
1	0.2	5~6	Brass disk, Φ15 mm × 1 mm
2	0.3	7~8	Brass disk, Φ30 mm × 1 mm
3	0.4	11~12	Brass disk, Φ30 mm × 2 mm

**Table 3:** Mean and Standard Deviation of Compressive Strength of Concrete.

Rubber Content (%)	20 °C	100 °C	200 °C	300 °C
0%	53.63 ± 0.47	50.95 ± 0.95	50.00 ± 1.31	48.98 ± 2.55
5%	46.36 ± 0.60	42.26 ± 0.92	39.06 ± 2.01	33.04 ± 2.06
15%	36.92 ± 0.76	31.63 ± 1.64	27.85 ± 1.79	21.77 ± 1.35
30%	29.18 ± 1.61	22.57 ± 1.65	19.05 ± 1.27	17.32 ± 3.65

Note: All values in the table are in MPa.

**Table 4:** Statistical results of splitting tensile strength of rubberized concrete.

	T (°C)	Splitting Strength (MPa)	Average Strength (MPa)	Decline (%)	Coefficient of Variation (%)
NC	20	6.98, 6.67, 6.08	6.58	-	6.8
	100	7.11, 6.42, 5.78	6.44	2.1	8.9
	200	5.24, 5.60, 5.57	5.47	16.9	3.2
	300	4.92, 4.78, 5.94	5.21	20.8	10.5
RC5	20	4.68, 5.85, 5.29	5.27	-	10.3
	100	4.44, 5.31, 5.06	4.94	6.3	8.5
	200	5.27, 4.61, 3.91	4.60	12.7	12.3
	300	3.41, 5.31, 4.17	4.30	18.4	18.7
RC15	20	3.90, 3.47, 3.58	3.65	-	5.8
	100	3.39, 2.96, 3.76	3.37	7.7	10.2
	200	3.65, 2.40, 3.23	3.09	15.3	18.9
	300	2.73, 2.79, 2.71	2.74	24.9	1.3

**Table 5:** SHPB Test parameters and results of NC.

	Air Pressure (MPa)	Temperature (°C)	Velocity (m/s)	Average Strain Rate (s^−1^)	Dynamic Strength (MPa)	Strain (×10^−6^)	DIF
NC	0.2	20	5.856	107.6	66.0 ± 2.9	1242	1.23
		100	5.842	104.3	65.8 ± 3.5	978	1.29
		200	5.847	96.9	67.4 ± 3.4	846	1.35
		300	5.796	100.5	67.3 ± 5.9	648	1.37
	0.3	20	7.492	143.5	113.5 ± 3.3	1758	2.12
		100	7.356	144.2	113.1 ± 7.6	2886	2.22
		200	7.659	140.0	110.5 ± 8.9	2658	2.21
		300	7.635	141.8	108.9 ± 11.2	3510	2.22
	0.4	20	11.716	173.5	143.7 ± 6.1	2142	2.68
		100	11.514	168.5	143.7 ± 7.0	2790	2.82
		200	11.447	174.3	139.4 ± 11.8	3408	2.79
		300	11.031	171.1	142.2 ± 15.3	3396	2.90

**Table 6:** SHPB Test parameters and results of RC5.

	Air Pressure (MPa)	Temperature (°C)	Velocity (m/s)	Average Strain Rate (s^−1^)	Dynamic Strength (MPa)	Strain (×10^−6^)	DIF
RC5	0.2	20	5.624	101.5	49.5 ± 4.1	2550	1.07
		100	5.658	97.9	56.4 ± 5.7	3012	1.34
		200	5.716	104.2	47.8 ± 5.6	2694	1.22
		300	5.622	98.2	45.5 ± 9.2	2688	1.38
	0.3	20	7.698	134.0	113.4 ± 2.7	3048	2.45
		100	7.608	141.4	131.5 ± 5.3	2736	3.11
		200	7.916	141.5	107.4 ± 3.8	3588	2.75
		300	7.910	135.4	100.5 ± 7.6	3588	3.05
	0.4	20	11.517	168.3	144.1 ± 10.1	3492	3.11
		100	11.507	177.4	152.2 ± 8.4	2148	3.60
		200	11.481	184.7	138.5 ± 14.5	3546	3.54
		300	11.547	178.1	128.5 ± 16.1	3318	3.89

**Table 7:** SHPB Test parameters and results of RC15.

	Air Pressure (MPa)	Temperature (°C)	Velocity (m/s)	Average Strain Rate (s^−1^)	Dynamic Strength (MPa)	Strain (×10^−6^)	DIF
RC15	0.2	20	5.815	112.4	43.6 ± 8.2	1878	1.18
		100	5.866	113.1	46.5 ± 4.1	1704	1.47
		200	5.897	107.4	41.0 ± 6.5	2814	1.47
		300	5.763	113.7	37.7 ± 10.4	2886	1.73
	0.3	20	7.560	149.7	73.5 ± 9.2	2676	1.99
		100	7.722	149.1	84.0 ± 11.1	2466	2.66
		200	7.800	142.9	72.5 ± 15.4	1962	2.60
		300	7.824	143.9	73.5 ±14.9	3858	3.37
	0.4	20	11.177	172.5	122.9 ± 13.0	5556	3.33
		100	11.503	177.1	134.1 ± 14.4	4050	4.24
		200	11.516	176.3	126.1 ± 15.8	5286	4.52
		300	11.173	173.5	116.8 ± 17.6	5070	5.36

**Table 8:** Energy dissipation parameters of concrete under dynamic impact loading.

	Average Strain Rate (s^−1^)	Energy Dissipation (kJ/m^3^)			
		20 °C	100 °C	200 °C	300 °C
NC	100.3	185.0	199.1	175.0	191.4
	142.4	915.7	438.9	723.6	583.1
	171.9	885.0	613.0	754.3	640.3
RC5	100.5	192.6	207.7	176.7	161.4
	138.1	505.2	667.6	485.6	579.2
	177.1	604.8	853.9	605.2	802.0
RC15	111.7	361.5	369.2	286.2	279.4
	146.4	471.9	468.4	438.5	327.0
	174.9	817.8	1078.0	779.8	780.9

## Data Availability

The original contributions presented in this study are included in the article. Further inquiries can be directed to the corresponding author.

## Abbreviations

The following abbreviations are used in this manuscript:

NC	Normal concrete
RC	Rubber concrete
SHPB	Split Hopkinson Pressure Bar
DIF	Dynamic Increase Factor
